# Development and Mechanical Characterization of Novel Gelatin–Fish Oil Composite Hydrogel for 3D Bioprinting

**DOI:** 10.3390/biomimetics11100704

**Published:** 2026-10-03

**Authors:** Djordje Milosevic, Marija Brankovic, Katarina Virijevic, Nikola Kotorcevic, Dusan Milojevic, Nevena Mihailovic, Nenad Grujovic, Fatima Zivic

**Affiliations:** 1 Faculty of Engineering, University of Kragujevac, Sestre Janjic 6, 34000 Kragujevac, Serbia; dj.miloshevich@gmail.com (D.M.); marija.brankovic@uni.kg.ac.rs (M.B.); msc.katarina.virijevic@gmail.com (K.V.); nikola.kotorcevic@uni.kg.ac.rs (N.K.); dusanmilojevic@uni.kg.ac.rs (D.M.); gruja@kg.ac.rs (N.G.); 2 Institute for Information Technologies, University of Kragujevac, Liceja Kneževine Srbije 1A, 34000 Kragujevac, Serbia; 3 Faculty of Science, University of Kragujevac, Radoja Domanovica 12, 34000 Kragujevac, Serbia; nevena.mihailovic@pmf.kg.ac.rs

**Keywords:** 3D bioprinting, gelatin-based bioink, AI-supported formulation design, multicomponent hydrogel, contact mechanics, spherical and flat indentation, ISO/WD TS 25812, ASTM F04.42, ASTM WK92859, UN SDG3

## Abstract

Gelatin-based hydrogels are attractive bioinks for extrusion bioprinting because they combine biocompatibility, tunable rheology, and the ability to incorporate bioactive compounds. In this study, a novel multicomponent gelatin hydrogel containing fish oil, Tween 80, lecithin, glycerin, psyllium husk, and ascorbic acid was initially designed using AI-supported formulation screening and subsequently optimized experimentally for extrusion-based 3D printing. Nine acellular hydrogel constructs were fabricated using printing speeds of 1, 2, and 5 mm/s and infill densities of 5, 15, and 30%. Optical imaging, storage stability, experimentally measured density, water-contact-angle measurements, ATR-FTIR spectroscopy, and compression testing using flat cylindrical and spherical indenters were employed to characterize the printed constructs. Mass losses exceeded 60% after 7 days and reached approximately 91% after 110 days. Measured densities ranged from 0.22 to 1.24 g/cm^3^ across samples. All samples were hydrophilic, with contact angles of 30.07–50.63°, and higher infill increased contact angle. ATR-FTIR showed similar chemical profiles, with no clear infill-related chemical changes, suggesting that mechanical differences mainly reflected physical architecture and heterogeneity. Flat indentation generally produced higher maximum loads, except for sample S6, which reached 396.24 N under spherical loading and had the highest density (1.24 g/cm^3^), suggesting that its exceptional mechanical response may be associated with local material compactness and structural heterogeneity. Stress-ratio analysis yielded a mean σr/σtrue value of 4.43 with a coefficient of variation of 97.41%, while Bland–Altman analysis showed a mean bias of 0.15 MPa and limits of agreement from −0.70 to 1.01 MPa, demonstrating that the two testing methods are not directly interchangeable. Overall, hydrogel composition, internal network structure, printing infill, and local contact mechanics influenced mechanical behavior, highlighting the need for reproducible, method-specific hydrogel characterization.

## 1. Introduction

3D bioprinting has enabled the fabrication of biomimetic scaffolds with a broad range of compositions for diverse biomedical applications [1,2]. Bioprinting has evolved to enable highly accurate 3D in vitro models and functional tissue constructs [3], as well as the precise incorporation and spatial organization of living cells within three-dimensional scaffolds, or their direct deposition as bioinks, with the long-term goal of fabricating functional tissues and ultimately transplantable organs [4]. Extrusion-based bioprinting is well suited for highly viscous bioinks [5]. However, significant challenges remain, primarily related to material selection and formulation design to ensure smooth extrusion, continuous filament formation during deposition, rapid recovery of structural integrity after extrusion, and retention of the prescribed geometry.

Recent studies have employed various material strategies in bioprinting to achieve optimal printability and dimensional accuracy, besides adequate mechanical strength. A photo- and thermo-responsive silk fibroin–iota-carrageenan system was developed to improve extrusion behavior and post-deposition stabilization through complementary physical and photochemical crosslinking mechanisms [6]. Gelatin and sodium alginate have been combined with decellularized extracellular matrix (dECM) to produce more tissue-relevant bioink [7]. The addition of graphene oxide and calcium phosphate has been investigated to improve the mechanical and functional properties of printed scaffolds based on GelMA bioinks [8]. Tuning composite blends for bioprinting was done with combinations of polyvinyl alcohol (PVA) and dECM to provide high mechanical strength along with specific biological cues [9]. All these studies demonstrated that material combinations are a challenging task but are essential to satisfy simultaneous requirements of optimal printability, biological activity, dimensional stability, and mechanical strength. Synthetic polymers enable better control over composition, degradation, and mechanical behavior but usually require biological functionalization. Natural materials can provide biologically relevant environments but commonly lack adequate mechanical properties and stability over time [10]. Nanomaterial reinforcements can add various properties, including functional ones, but their use must consider cytocompatibility besides other influences [11]. Consequently, composite bioinks have emerged as an effective strategy for overcoming the inherent trade-offs among printability, mechanical integrity, and biological performance, enabling the development of multifunctional materials suitable for extrusion-based bioprinting applications [2,12].

Gelatin-based systems are commonly investigated because of their biocompatibility, biodegradability, and ability to form gel-like structures suitable for scaffold fabrication [2]. Nevertheless, additional components are required to improve flexibility, stability, viscosity, and overall printability [5]. In this context, plasticizers, natural fibers, emulsifiers, antioxidants, and bioactive lipid components may be incorporated to enhance the functional and structural properties of the printed material. Glycerin acts as an effective plasticizing agent by reducing intermolecular interactions and increasing polymer chain mobility, thereby improving the flexibility, ductility, and structural integrity of polymeric materials while enhancing the printability and durability of 3D-printable formulations [13]. The particle size of psyllium husk significantly influences gel strength, water-holding capacity, and rheological properties, supporting its use as a natural viscosity modifier and structural stabilizer in hydrogel formulations [14]. Fish oil exhibits anti-inflammatory, antioxidant, and regenerative properties, which make it an attractive bioactive component for tissue engineering applications [15]. The combined use of Tween 80 and soy lecithin produces stable nanoemulsions with improved encapsulation efficiency and emulsion stability, supporting their application as complementary emulsifiers in lipid-containing hydrogel systems [16]. Ascorbic acid exhibits antioxidant activity, has an important role in collagen synthesis, and has the ability to protect biomaterials and biological systems from oxidative degradation [17].

Printability and dimensional shape retention alone are insufficient to establish the practical suitability of a hydrogel-based printed scaffold. Hydrated polymer networks commonly exhibit nonlinear, time-dependent, and formulation-dependent deformation, while the stiffness of a printed sample is additionally influenced by crosslinking conditions, sample geometry, porosity, hydration state, loading rate, and the strain interval selected for modulus calculation [18]. Beyond their role as printable biomaterials, functionalized polymer hydrogels are increasingly recognized as versatile platforms for biomedical, therapeutic, and advanced delivery applications [19]. Their chemical composition, network architecture, crosslinking density, porosity, mechanical properties, and responsiveness to external or physiological stimuli can be tailored to achieve application-specific functions, including controlled or targeted delivery of active compounds, tissue interaction, and environmentally responsive behavior. In this context, the development of multicomponent hydrogel systems enables the combination of complementary functionalities, such as structural support, hydration, rheological control, lipid incorporation, and the delivery of bioactive molecules. Therefore, the design of customizable hydrogel matrices provides a broader framework for the development of advanced formulations beyond printability alone, supporting their potential use in responsive and application-specific delivery systems [19].

Compression testing is widely used to characterize the load-bearing response of printed hydrogels and to determine whether material composition and fabrication parameters provide mechanically stable structures [18]. 3D printed hydrogel systems are highly sensitive to both formulation and processing conditions, and time-dependent degradation must be considered from the aspect of compressive modulus [20]. Mechanical moduli are dependent on the material properties, such as hydrogel concentration, and also on printing parameters, where advanced computational models can be used to optimize and predict suitable formulations and processing conditions [21]. The addition of nano-phases as hydrogel reinforcements can further tailor the final mechanical properties, such as stiffness, yield strength, and resistance to large deformation [22,23]. Mechanical characterization of the developed new hydrogel formulations is of the utmost importance, since the mechanical stiffness of biomaterial substrates has a critical role in regulating cell behavior, differentiation, and tissue regeneration through dynamic cell–material interactions [24].

The need for comprehensive and reproducible hydrogel characterization has become increasingly important in light of ongoing international standardization initiatives, including ISO/TC 276 and ASTM F04.42, which are developing standardized methodologies for evaluating hydrogel performance, printability, quality control, and mechanical properties for biofabrication and regenerative medicine applications [25]. ISO/WD TS 25812, currently under development by ISO/TC 276, seeks to standardize the performance evaluation and quality assessment of hydrogels for bio-device fabrication, including bioinks [26]. ASTM WK92859 is an ongoing work item aimed at standardizing the characterization of bioink printability in extrusion bioprinting, including measurements of extrusion pressure, filament fidelity, strand collapse, print resolution, shape retention, and reproducibility [27]. These are currently measured differently in almost every laboratory. In May 2025, ASTM organized a workshop on the characterization of hydrogel medical products to identify gaps in existing standards and prioritize harmonized methods for mechanical, physicochemical, morphological, degradation, and application-specific testing [28]. Their announcement notes that there are still relatively few ASTM procedures specifically addressing hydrogel medical products, and one goal is to identify test methods suitable for future standardization.

Although gelatin-based bioinks have been extensively studied, limited information is available regarding gelatin systems simultaneously incorporating omega-3-rich fish oil and psyllium husk as rheological modifiers. Furthermore, the influence of printing parameters on the mechanical performance and structural stability of such composite hydrogels remains insufficiently investigated. This study has developed a bio-based 3D printable acellular gelatin-based hydrogel–emulsion formulation containing gelatin, fish oil, Tween 80, lecithin, glycerin, psyllium husk, and ascorbic acid. Gelatin was selected as the primary matrix-forming component, while the remaining constituents were incorporated to modify emulsion stability, flexibility, viscosity, oxidative stability, and extrusion behavior. The objective of this study was to develop a formulation suitable for extrusion-based three-dimensional bioprinting and to evaluate its printability, short-term morphological stability, and mechanical response as a function of the selected printing parameters. The present multicomponent lipid–hydrogel formulation was conceived not only from the perspective of extrusion-based printability, but also as an initial platform in which the matrix composition could potentially be further tailored to control structural properties, stability, and the incorporation or delivery of functional components.

## 2. Bioprinting

Bioprinting has been developed focusing on the possibility of incorporating living cells in 3D printed scaffolds, or directly printing them, aiming for 3D printing of living organs [4]. An overview of the five stages in the development of 3D printing for biomedical applications is presented in Table 1, from non-living medical models and implants to advanced bioprinting of living cells and organoid-based in vitro systems [12]. However, suitable bioinks for bioprinting are still widely lacking. Bioinks for 3D printing of acellular scaffolds, namely those that do not contain cells, have been studied to enable printable and sufficiently strong mechanical scaffolds to support a range of applications [2]. A general overview of currently available bioinks is given in Table 2 [12].

## 3. Materials and Methods

### 3.1. AI-Supported Design of the Formulation Concept

The development of multifunctional hydrogels for extrusion-based bioprinting involves a complex, multidimensional design space in which formulation components and their concentrations must be balanced to achieve appropriate rheological behavior, printability, structural stability, mechanical performance, and application-specific functionality. Recent advances in model-based, machine-learning, and artificial intelligence (AI)-assisted approaches have therefore highlighted the potential of computational tools to accelerate hydrogel design, formulation screening, and multi-parameter optimization [29].

The use of large language models (LLMs) for materials research and discovery is an emerging research direction, with recent studies highlighting their potential for hypothesis generation, materials-information extraction, candidate generation, and materials discovery, while also emphasizing the limitations of general-purpose models and the need for domain-specific materials-science LLMs [30,31]. Natural language processing (NLP) and LLM-based approaches are increasingly being explored for automated materials-information extraction, materials discovery, property and synthesis-route analysis, and, in more advanced workflows, autonomous and closed-loop materials research [32]. In the present study, an LLM was used only as a formulation ideation and hypothesis-generation tool to propose an initial multicomponent hydrogel formulation. The AI-generated output was not treated as experimental evidence, and all subsequent formulation selection and optimization were based on experimental observations and characterization.

In this context, NLP technology through ChatGPT 4.0 was used as an AI-supported ideation tool to generate an initial formulation concept based on predefined material and functional requirements [33]. The objective was to identify a potential composition of a shear-thinning hydrogel [5] suitable for extrusion-based printing, a formulation based predominantly on biocompatible, naturally derived or bio-based components and with potential application as a cosmetic patch. The predefined requirements included a gelatin-based matrix incorporating deuterated polyunsaturated fatty acids (D-PUFAs) and hyaluronic acid, together with sufficient consistency for controlled extrusion, continuous filament deposition, short-term shape retention after printing, and stable incorporation of a lipid phase.

The initially AI-suggested formulation comprised gelatin (fish or vegan), D-PUFA ethyl ester, polysorbate 80 (Tween 80), hyaluronic acid (150 kDa), glycerin, ascorbic acid, and distilled or rose water, together with a proposed preparation procedure. Based on the current research landscape, a hydrogel based on gelatin, fish oil, hyaluronic acid, and D-PUFAs is very important in research and cosmetic applications [34,35], which was our initial requirement for the NLP technology to design 3D printable hydrogel. A hybrid hydrogel combining fish gelatin, fish oil, hyaluronic acid, and deuterated polyunsaturated fatty acids (D-PUFAs) is highly important and represents a cutting-edge advancement in both biomedical research and advanced cosmetic applications. By fusing structurally supportive marine biopolymers with advanced isotope-reinforced lipids, this specific multi-component system targets the native extracellular matrix (ECM) while offering very high protection against oxidative stress and aging. In tissue engineering and dermatological research, this hydrogel acts as a highly functional, biocompatible scaffold. Combining fish gelatin and hyaluronic acid creates a network that effectively mimics native human skin. Because it utilizes marine-derived gelatin rather than mammalian sources (porcine or bovine), it carries zero risk of zoonotic disease transmission (like BSE). Normal polyunsaturated fatty acids (PUFAs) in fish oil are highly prone to lipid peroxidation (autoxidation) from UV and oxygen, turning into toxic by-products that damage cells. D-PUFAs replace hydrogen atoms with deuterium at vulnerable carbon sites. This utilizes the kinetic isotope effect to dramatically slow down oxidation, stopping cellular aging and lipid damage at a foundational level. Researchers use these composite networks to create artificial 3D skin models for toxicity testing, avoiding the need for animal testing while accurately capturing skin cellular behavior.

In advanced cosmetics and “cosmeceuticals,” each component serves a precise synergistic role. Gelatin provides a structural matrix and cell-adhesion properties, forming a smooth, flexible film on the skin to seal in active nutrients. Hyaluronic Acid (HA) acts as a deep humectant and hydration regulator, retaining immense moisture, plumping the skin and smoothing fine lines. Fish Oil acts as a supplier of necessary fatty acids (EPA/DHA), strengthens the skin barrier, reduces inflammation, and treats dry skin. D-PUFAs act as a stabilizer and ultimate free-radical shield, preventing the formula itself from going rancid while actively protecting skin cell membranes from UV photo-aging. The cross-linked hydrogel network stabilizes the fragile oils, providing a sustained, controlled release of localized nutrition to the epidermis. Standard fish oil cosmetics smell bad over time due to oxidation. Incorporating D-PUFAs chemically reinforces the formula, providing an inherent, non-antioxidant method to neutralize reactive oxygen species (ROS) damage triggered by sun exposure.

The use of AI at this stage was intended to facilitate hypothesis generation and the exploration of potential component combinations rather than to predict the final material properties or replace experimental formulation development. This approach is consistent with emerging AI-supported strategies for hydrogel design, in which computational methods can assist in navigating complex formulation spaces, identifying candidate compositions, and supporting iterative optimization, while experimental validation remains essential [33,36,37].

Importantly, the AI-generated output was treated only as an initial conceptual framework and not as experimental evidence or validation of the suitability, printability, stability, or biological performance of the proposed material. Several formulations with different component compositions and concentrations were subsequently prepared and evaluated experimentally. All compositional modifications and selection of the final formulation were based on experimental observations, the intended functional contribution of the individual components, extrusion behavior, printability, and structural stability. Thus, the final hydrogel composition and preparation protocol were established through iterative experimental refinement, with AI serving solely as a supporting tool during the initial formulation design stage.

### 3.2. Preparation of Samples and Experimental Refinement

Several formulations with different component compositions and concentrations were prepared and evaluated during the experimental refinement process. The compositions of the initial, refined, and final formulations are presented in Table 3.

The first experimental formulation contained an agar-based gelling blend, omega-3-rich fish oil, polysorbate 80 (Tween 80), hyaluronic acid (150 kDa), glycerin, 70% (*v*/*v*) ethanol, and distilled water. The agar-based blend served as the principal structuring component of the aqueous phase, while the fish oil represented the lipid phase. Polysorbate 80 was included to facilitate dispersion of the hydrophobic lipid phase within the aqueous system, whereas glycerin was incorporated as a plasticizing and humectant component. Ethanol was used in a small amount to facilitate initial handling and dispersion of the lipid phase.

Preliminary testing showed that the initial formulation containing 2.5 g of the agar-based gelling blend had insufficient viscosity and consistency for reliable extrusion and post-deposition shape retention. Therefore, its amount was increased to 3.5 g in the subsequent formulation. Hyaluronic acid was included in the initial formulation; however, the available quantity was insufficient to allow its consistent use throughout the complete formulation-development study. It was therefore excluded from subsequent formulations rather than being evaluated under inconsistent experimental conditions, and its specific contribution to the final material properties was not assessed in the present study.

To further improve the homogeneity and stability of the lipid-containing system, soy lecithin was introduced as an additional emulsifying component. Psyllium husk powder was also incorporated as a viscosity-modifying and structuring component. The addition of 0.4 g psyllium increased the consistency and cohesiveness of the formulation and improved its behavior during extrusion. Its amount was subsequently increased to 0.5 g in the final formulation to further improve extrusion consistency and post-deposition shape retention. L-ascorbic acid was introduced as an antioxidant component to reduce oxidative processes in the lipid-containing formulation.

The final composition was therefore selected through iterative experimental refinement rather than formal numerical optimization. Selection was based on macroscopic formulation homogeneity, stability of the dispersed lipid phase, consistency, extrusion behavior, continuity of the deposited filament, and short-term retention of the printed geometry.

No chemical or enzymatic crosslinking procedure was employed during formulation preparation. Consequently, the effects of compositional changes are interpreted in terms of changes in physical network formation, non-covalent intermolecular interactions, formulation viscosity and cohesion, and lipid-phase stability rather than changes in covalent crosslinking density.

### 3.3. Preparation of the Printable Hydrogel Formulation

All samples used in the subsequent experiments were prepared using the final printable hydrogel formulation presented in Table 3. Distilled water (10 mL) was transferred into a glass beaker and maintained in a water bath at 40 °C throughout the preparation procedure. The agar-based gelling blend (3.5 g) was gradually added to the distilled water under continuous manual stirring. Stirring was maintained throughout the preparation process to promote uniform incorporation of all components.

Omega-3 fish oil (4 µL) was premixed with 70% (*v*/*v*) ethanol (2 µL) and gradually added to the aqueous mixture under continuous stirring. Polysorbate 80 (Tween 80; 4 µL) was subsequently added, followed by soy lecithin (4 µL), to facilitate dispersion and stabilization of the lipid phase within the aqueous matrix.

Before incorporation into the formulation, psyllium husks were mechanically milled using an IKA^®^-WERKE M20 laboratory mill (Staufen im Breisgau, Germany) and subsequently passed through a 1.16 mm mesh sieve to reduce the size of larger particles and agglomerates and to facilitate more uniform dispersion. The prepared psyllium powder (0.5 g) was then gradually incorporated into the mixture under continuous manual stirring until a macroscopically homogeneous consistency was obtained. Glycerin (4 µL) and L-ascorbic acid (2 mg) were subsequently added, and mixing was continued until all components were uniformly incorporated.

Following preparation, the hydrogel formulation was conditioned at 5 °C for 30 min and subsequently transferred into the syringe of the 3D printer. Three independent batches of the final formulation were prepared, and all 168 analyses were performed in triplicate.

### 3.4. Fabrication of Acellular Constructs by Extrusion-Based 3D Printing

**Figure 1:**
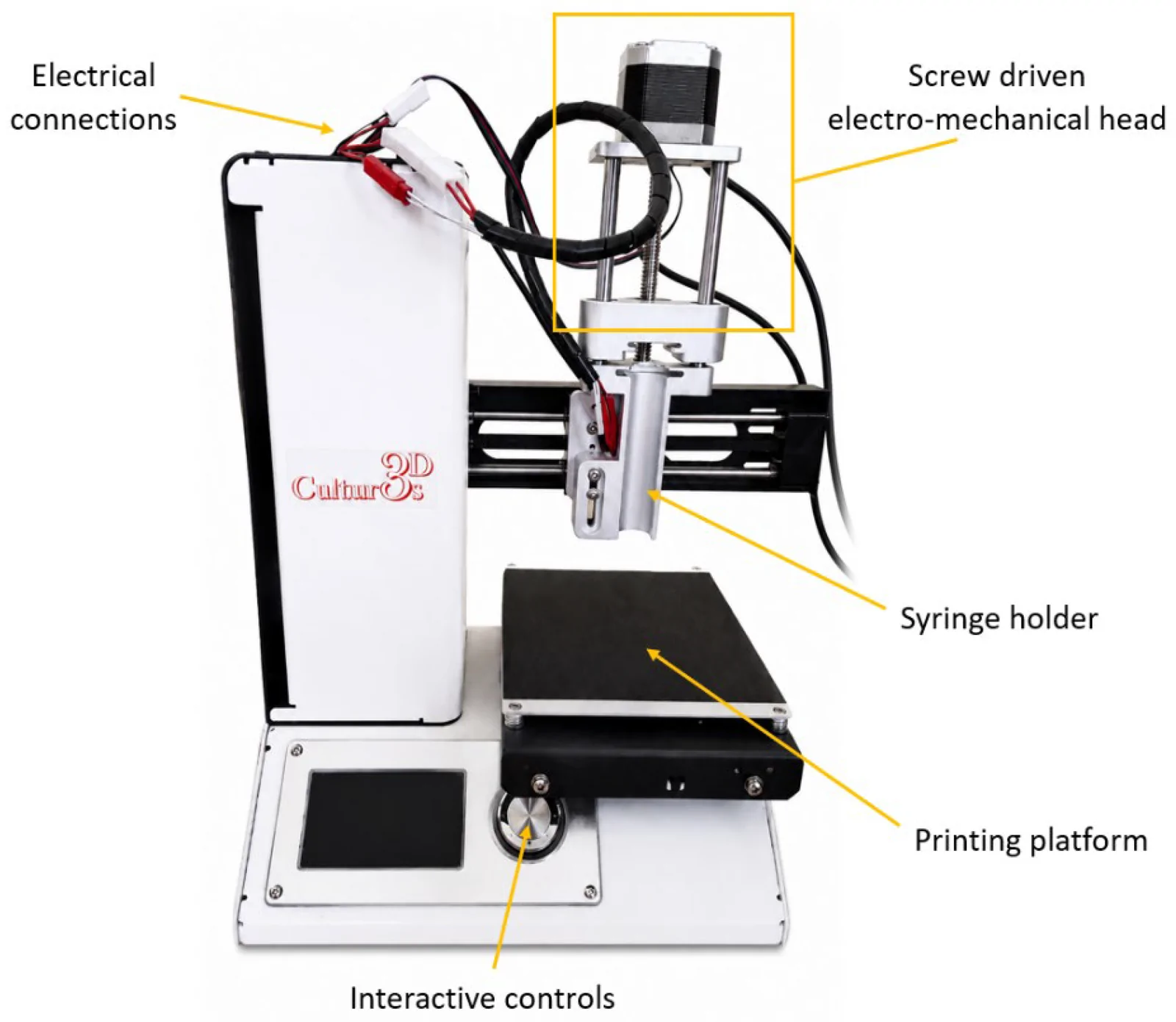
Main components of the Tissue Scribe 3D Cultures bioprinter (3D Cultures, Inc., Philadelphia, PA, USA).

A Tissue Scribe 3D Cultures extrusion-based 3D printing system was used for hydrogel construct fabrication (Figure 1). Following conditioning at 5 °C for 30 min, the final hydrogel formulation was transferred into a syringe and mounted in the printing system. The material was extruded through a needle with an inner diameter of 1.28 mm and deposited layer by layer onto the printing platform according to the predefined printing parameters.

To evaluate the influence of printing conditions on the properties of the fabricated constructs, three printing speeds (1, 2, and 5 mm/s) were combined with three infill densities (5, 15, and 30%), resulting in nine printing conditions (samples S1–S9), as summarized in Table 4. For all samples, the layer height was maintained at 0.1 mm, the number of perimeter walls (shells) was set to 0, and a line-based infill pattern was used. All samples were fabricated using the same final hydrogel composition and identical nominal dimensions.

**Figure 2:**
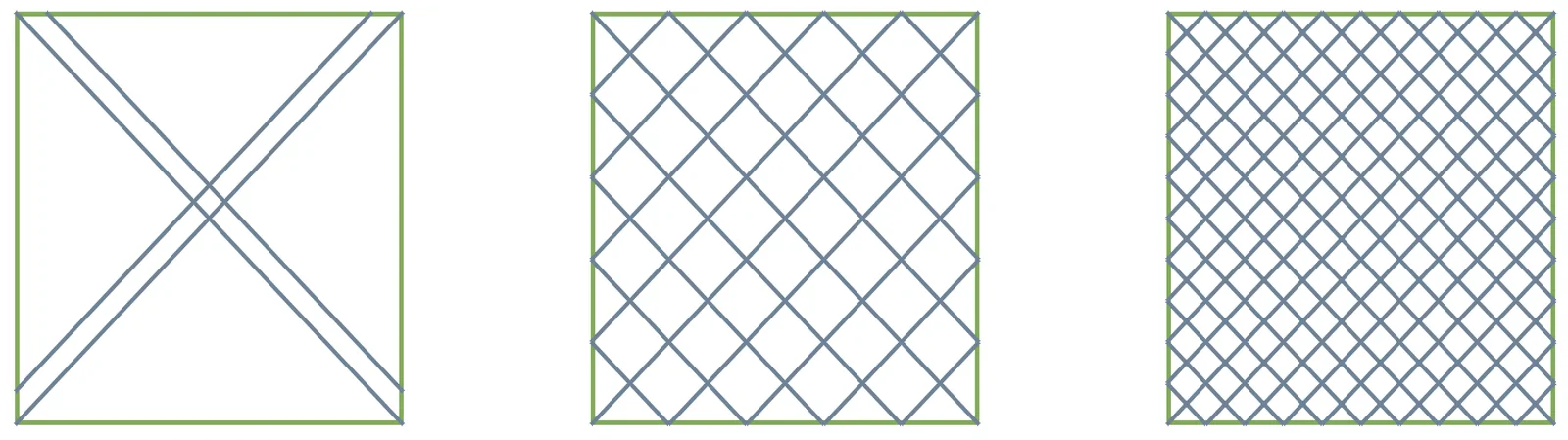
Schematic representation of line-based infill geometries. (**Left**) to (**right**): 5% infill/1 mm/s, 15% infill/1 mm/s, and 30% infill/1 mm/s.

A total of nine samples of the final hydrogel formulation, all with identical dimensions and a line-based infill pattern, were fabricated for further mechanical testing, using three different printing speeds and infill densities. Dimensions of the samples were 30 mm in length, 30 mm in width, and 10 mm in height. A schematic overview of the sample geometries corresponding to the different infill densities is shown in Figure 2.

### 3.5. Sample Characterisation

#### 3.5.1. Optical Microscopy

The printed hydrogel constructs were examined using a BTC STM8T trinocular stereo microscope (BTC, Budapest, Hungary) immediately after 3D printing and again after 7 days of storage at 4 °C. Microscopy was used to qualitatively assess preservation of the printed geometry and visible structural changes during storage.

#### 3.5.2. Theoretically Calculated and Experimentally Measured Densities

The density of the samples was theoretically calculated and also experimentally measured. Total hydrogel density was theoretically determined as 1.40 g/mL from the total mass (≈14.02 g based on the composition of the formulation) and assuming the final hydrogel volume is approximately 10.0 mL.

Density was experimentally measured 7 days after fabrication using Archimedes’ principle, employing dry sand as the displacement medium to prevent swelling of the hydrogel samples.

#### 3.5.3. Theoretical Mass Estimation, Experimentally Measured Mass, and Mass Loss

The mass of the samples was theoretically calculated and also experimentally measured (immediately after fabrication, 3, 7, and 110 days afterwards) to analyse mass loss. The theoretical estimate of the printed hydrogel mass was based on sample dimensions (30 × 30 × 10 mm), printing parameters (Infill percentage, such as 5%; number of perimeters − 0), and hydrogel density. Total sample volume is assumed to be V = 30 × 30 × 10 = 9000 mm^3^, or 9.0 mL. With 0 perimeters, only the infill contributes material, which, in the case of material printed at 5% infill is:

V_printed_ = 9.0 × 0.05 = 0.45 mL (1)

from where the printed mass can be calculated as:

m = 0.45 × 1.40 = 0.63 g(2)

Accordingly, the printed mass for each of the infill percentages (5, 15, 30%) can be calculated and is shown in Table 5. Experimentally measured mass immediately after fabrication and after 3, 7, and 110 days of storage in dry air at room temperature (25 °C) is also given in Table 5.

Mass loss was calculated after 3, 7, and 110 days, from experimentally measured sample mass results, to evaluate the stability parameter for hydrogel samples, according to Equation (3):

Mass loss (%) = (W_0_ − W_t_)/W_0_ × 100 (3)

where W_0_, initial sample mass, W_t_, sample mass after storage time t.

#### 3.5.4. Surface Wettability and Contact Angle Measurement

**Figure 3:**
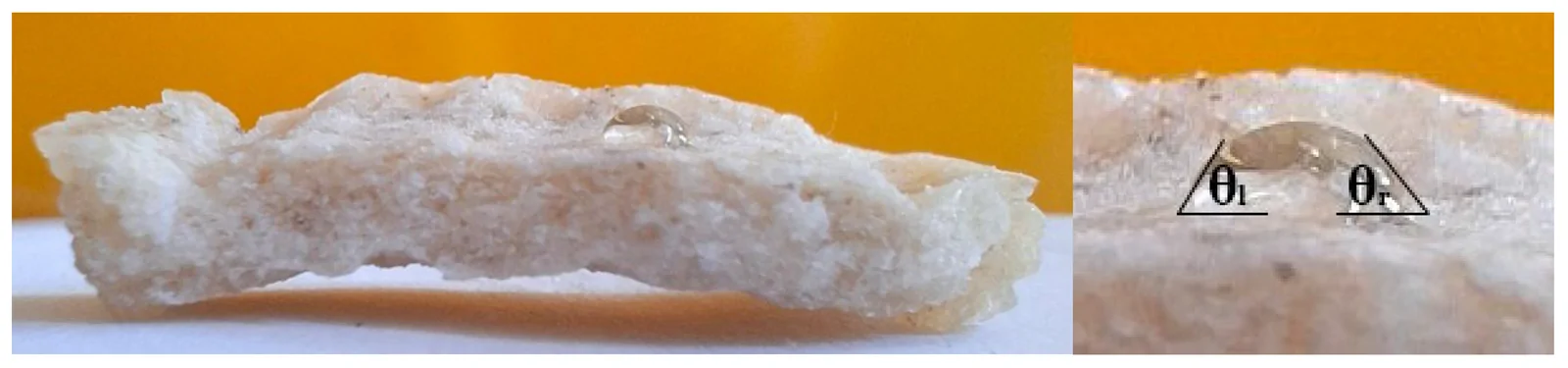
(**Left**): sample S6 with a 5 µL distilled water droplet deposited on the surface for wettability assessment; (**Right**): contact angle measurement on the left (θ_L_) and right (θ_R_) sides of the water droplet deposited on S6.

Wettability describes the ability of a liquid to spread over and maintain contact with a solid surface and is commonly assessed through water contact angle measurements. A lower contact angle indicates greater wettability and a more hydrophilic surface, whereas a higher contact angle indicates reduced wettability and a more hydrophobic surface. For wettability assessment, a 5 µL droplet of distilled water was deposited on the upper surface of each sample, as shown in Figure 3 (left). Three separate positions were evaluated on each sample. Side-view images of the droplets were analyzed. The apparent contact angle (θ) formed at the solid-liquid-air interface was measured on the left (θL) and right (θR) sides of each droplet, as illustrated in Figure 3 (right). Because the sample surfaces were rough and porous, the measured values were treated as apparent contact angles.

The mean contact angle for each individual droplet (θLR) was calculated according to Equation (4):

θLR = (θL + θR)/2(4)

Subsequently, the average contact angle for each sample (θavg) was calculated from the three θLR values obtained at the three different measurement positions according to Equation (5):

θavg = (θLR1 + θLR2 + θLR3)/3(5)

The mean contact angle per sample (θavg) was used as the primary indicator of surface wettability, with lower values corresponding to greater wetting by water.

#### 3.5.5. ATR-FTIR Analysis

The chemical characteristics of the printed hydrogel constructs were evaluated by attenuated total reflectance Fourier-transform infrared (ATR-FTIR) spectroscopy using a Thermo Scientific Nicolet 6700 FT-IR spectrometer (Thermo Fisher Scientific Inc., Waltham, MA, USA) equipped with an ATR accessory. Measurements were performed on all nine printed samples (S1–S9) after drying (41 days after sample fabrication). Spectra were recorded over the wavenumber range of 4000–400 cm^−1^. The spectra were evaluated to identify characteristic absorption bands and possible differences in molecular interactions among the printed samples.

#### 3.5.6. Compressive Testing

All samples were tested under compressive loads, 24 h after fabrication, using a Brookfield CT3 universal mechanical tester. The tool speed was 1 mm/s with target depth set to 3 mm. Testing of the samples under compression was realized with two different indenters: (1) spherical indenter TA43 (sphere diameter D = 25.4 mm) and (2) flat cylindrical indenter TA25/1000 (cylinder diameter at flat end D = 50.8 mm, length L = 20 mm). The end of the compressive test was defined by the same displacement reached for all samples of 3 mm.

## 4. Results and Discussion

The transition from the initial experimental formulation to the refined and final formulations involved systematic modification of the relative amounts and types of network-forming and functional components (Table 3). The most notable change was the increase in the agar-based gelling blend from 2.5 to 3.5 g, which was expected to increase the concentration of the continuous polymer phase and promote a denser physically associated network. In parallel, soy lecithin, psyllium husk powder, and L-ascorbic acid were introduced into the refined formulation, while hyaluronic acid was excluded. The incorporation of psyllium, followed by its increase from 0.4 to 0.5 g in the final formulation, introduced an additional polysaccharide-rich component capable of influencing water retention, chain interactions, and the rheological behavior of the multicomponent system. Soy lecithin was introduced to improve the dispersion and stabilization of the lipid-containing phase, whereas the removal of hyaluronic acid simplified the formulation and altered the balance between the different hydrophilic polymeric components. Thus, although no deliberate chemical crosslinking was applied, changes in polymer concentration and composition were expected to modify the microstructure and physical interaction network through changes in polymer–polymer interactions, hydrogen bonding, chain entanglement, and water binding. However, detailed characterization of the resulting microstructure and molecular interaction network would be required to directly confirm these mechanisms. Similar relationships between variations in natural polymer composition, microstructural organization, intermolecular interactions, and the resulting engineering properties have been reported for multicomponent hydrogel systems [38]. Accordingly, the progression of formulations in Table 3 should be considered an iterative compositional optimization in which the initial AI-supported formulation served as a starting hypothesis, while subsequent modifications were guided by experimental observations of printability, handling, and structural behavior. This interpretation is consistent with recent studies demonstrating that systematic variation in the composition of natural polymer-based multicomponent hydrogels can modify their microstructural organization, intermolecular interaction networks, and resulting engineering properties [38].

**Figure 4:**
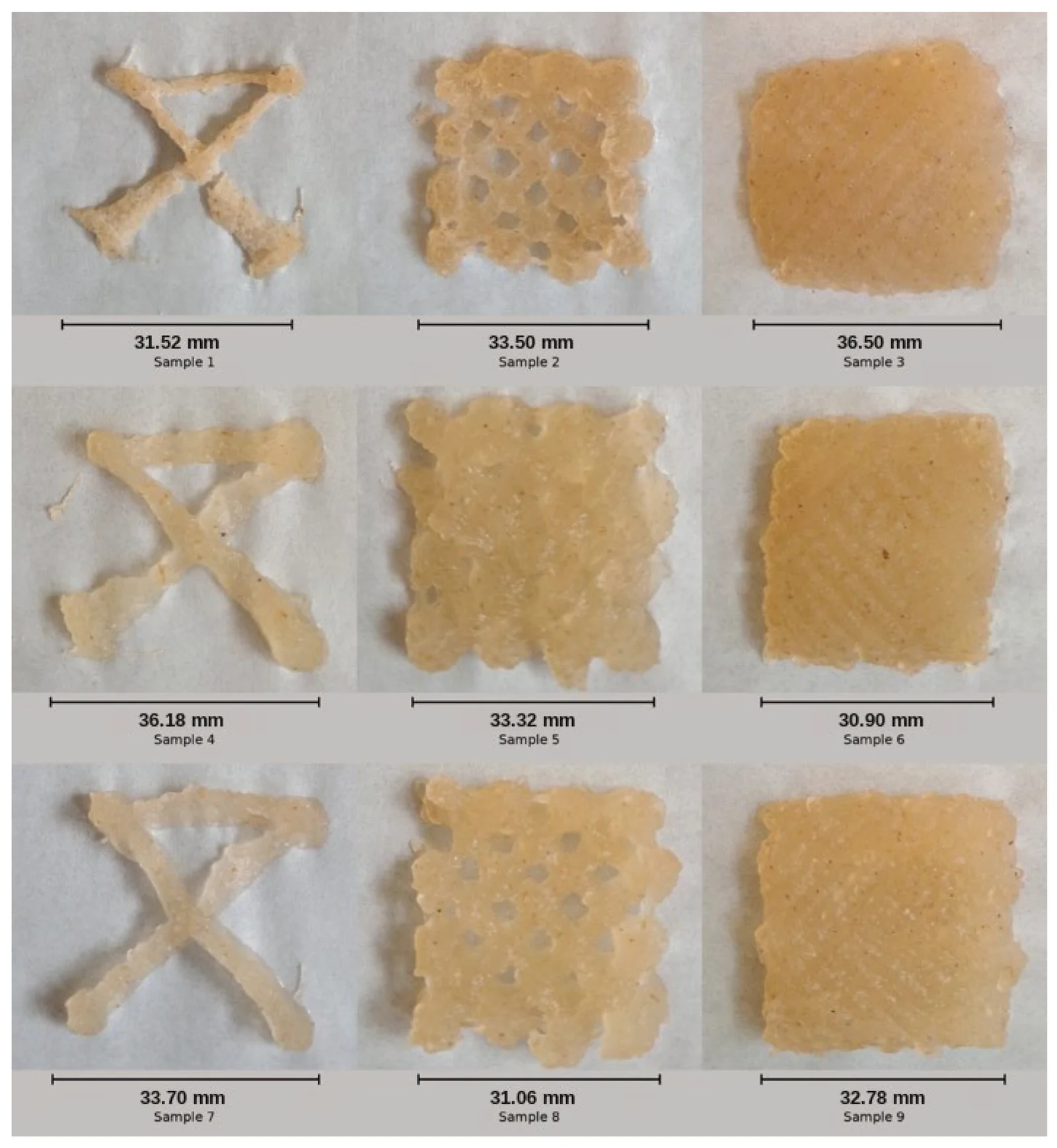
Optical images of all samples immediately after the fabrication.

Optical images of the samples immediately after the 3D printing are shown in Figure 4.

**Figure 5:**
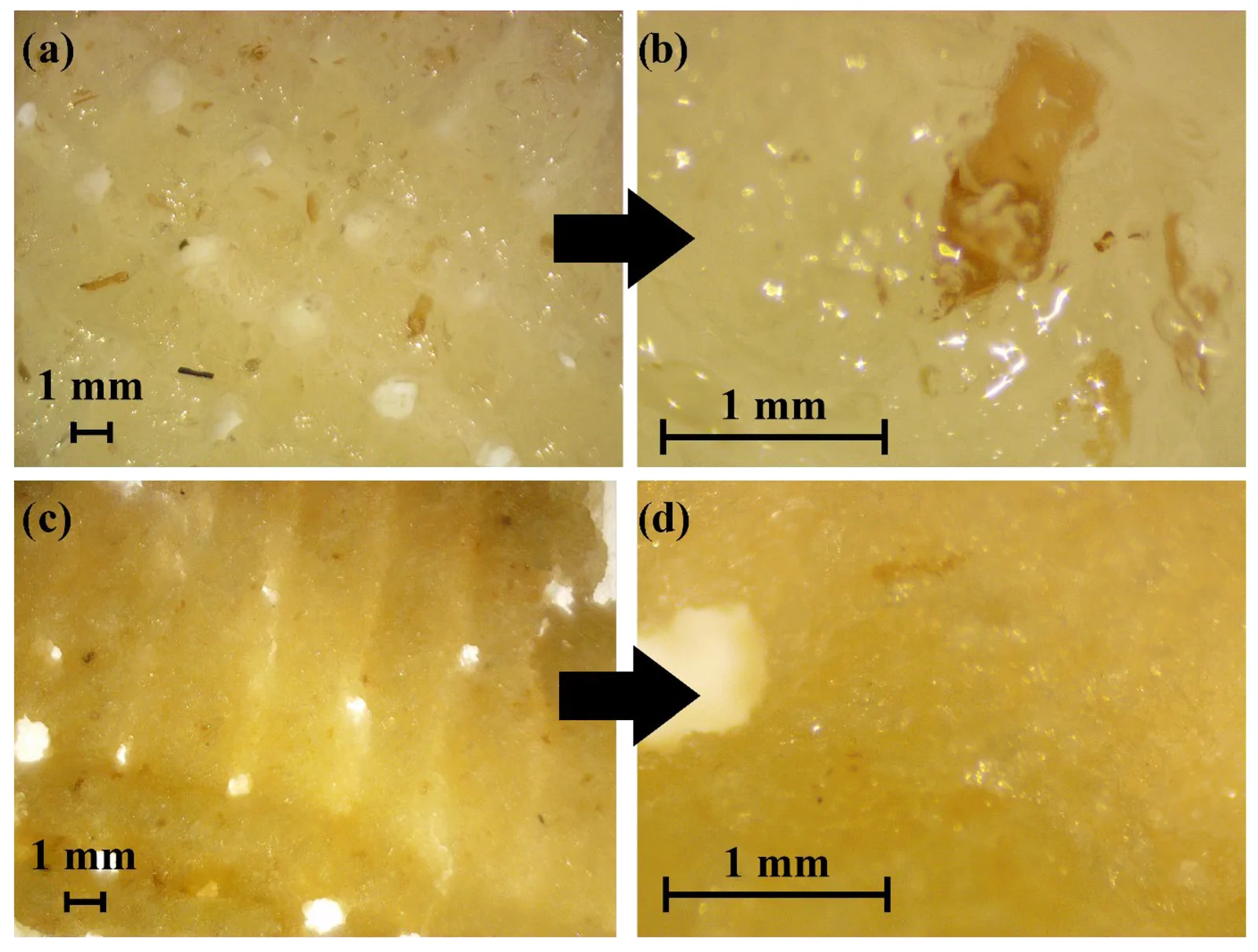
Optical images of the sample S3 immediately after 3D printing (top images (**a**,**b**)) and after one week (bottom images (**c**,**d**)).

**Figure 6:**
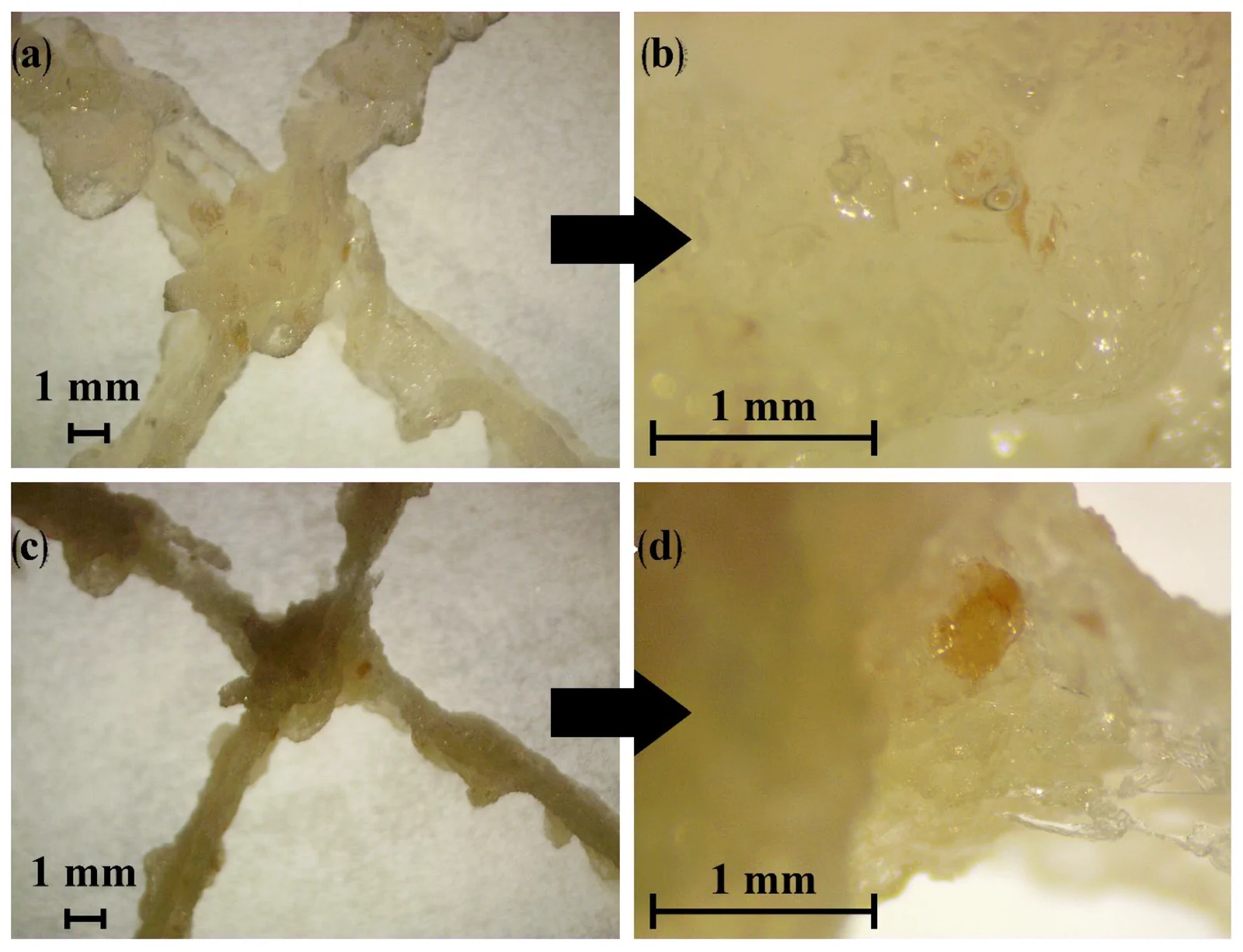
Optical images of the sample S4 immediately after 3D printing (top images (**a**,**b**)) and after one week (bottom images (**c**,**d**)).

**Figure 7:**
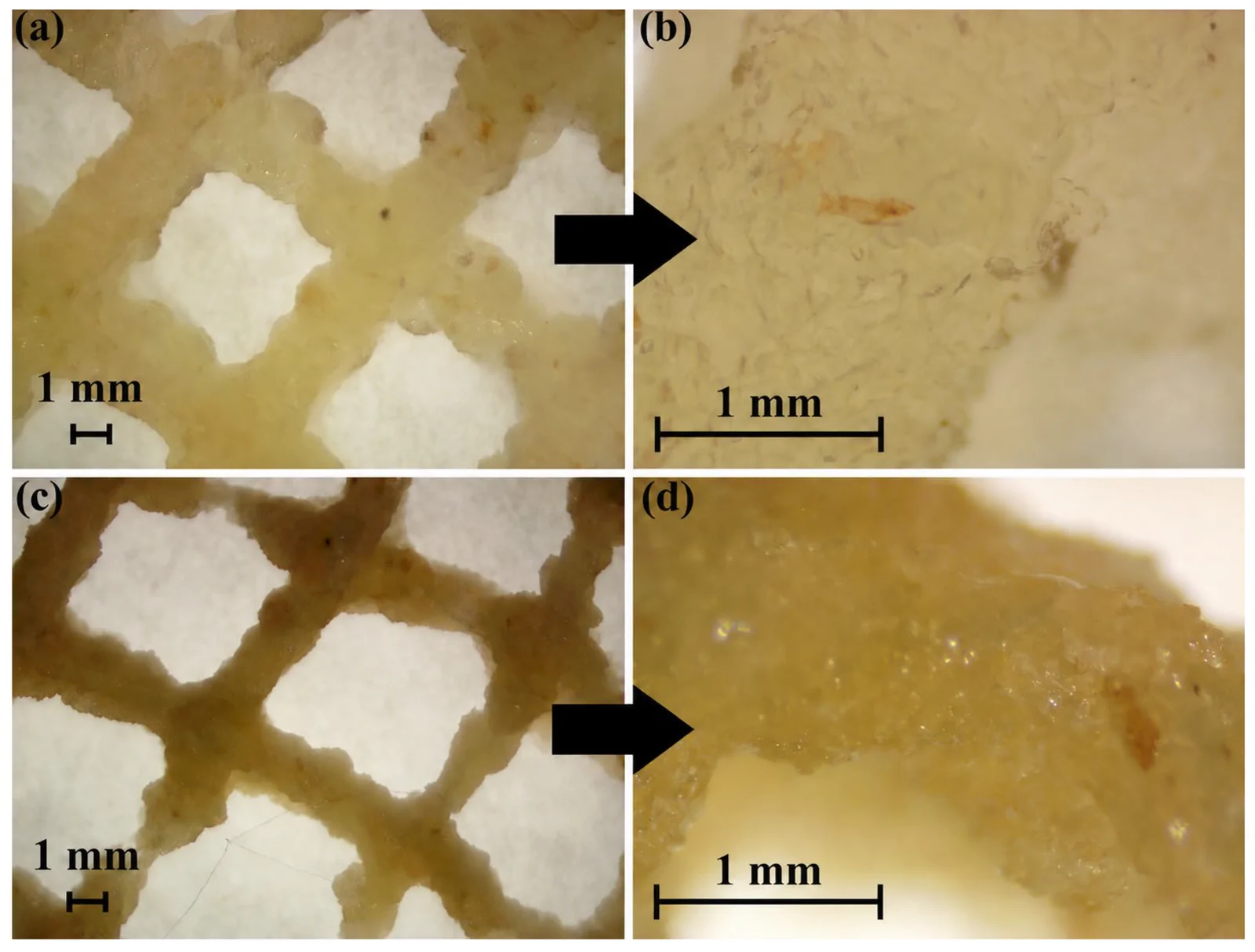
Optical images of the sample S5 immediately after 3D printing (top images (**a**,**b**)) and after one week (bottom images (**c**,**d**)).

Enlarged optical images of the samples immediately after the 3D bioprinting and after one week are shown in Figure 5, Figure 6 and Figure 7, for samples stored in a refrigerator. Clear loss of volume was observed after one week, along with a color change. This can be attributed to the drying process of the liquid phase. Also, it led to the change of samples from a fully elastic state immediately after fabrication to a rather brittle state after one week.

The developed formulation represents a multicomponent hydrogel–emulsion system composed of gelatin, fish oil, emulsifiers (Tween 80 and lecithin), glycerine, psyllium husk, ascorbic acid, and water. This combination resulted in a structurally stable and biocompatible material with properties suitable for potential biomedical applications.

Gelatin was selected as the primary matrix-forming component due to its well-established functionality and versatility. As a denatured form of collagen, gelatin is widely used in pharmaceutical, food, and cosmetic applications because of its gel-forming ability, low antigenicity, and excellent biocompatibility. It is available in high-quality grades and can be readily modified through various physical and chemical approaches [39]. These characteristics make gelatin particularly suitable for designing delivery systems aimed at protecting bioactive compounds and enhancing their efficacy. Moreover, its tunable mechanical properties potentially enable the fabrication of functional 3D scaffolds for tissue regeneration [40].

The incorporation of fish oil introduced a dispersed lipid phase within the hydrogel network. In addition to its structural role in emulsion formation, fish oil contributes bioactive lipids with well-documented anti-inflammatory properties [41]. The fatty acids present in fish oil are key components of neural and retinal membranes, and their presence within the matrix may contribute to reduced risks of atherosclerosis, chronic inflammation, and cognitive decline [42].

A homogeneous dispersion of the lipid phase within the gelatin matrix was achieved through the combined action of emulsifiers, effectively preventing phase separation during processing. From a biological perspective, inflammation is a complex response involving the production of cytokines, reactive oxygen species (ROS), adhesion molecules, and eicosanoids derived from arachidonic acid [43]. Long-chain polyunsaturated fatty acids modulate this response by inhibiting arachidonic acid metabolism [44], thereby shifting eicosanoid production toward omega-3-derived compounds with anti-inflammatory activity. Additionally, eicosapentaenoic acid (EPA) and docosahexaenoic acid (DHA) regulate gene expression related to inflammatory and atherogenic pathways [45]. These effects are further supported by a meta-analysis of 31 randomized controlled trials, which demonstrated a significant reduction in circulating inflammatory markers following omega-3 supplementation [46].

Emulsion stability was further enhanced by the synergistic action of Tween 80 and lecithin. Tween 80, a non-ionic surfactant, is characterized by low toxicity, good biocompatibility, and cost-effectiveness, and it plays a key role in reducing interfacial tension between hydrophilic and lipophilic phases [47]. Lecithin, as a phospholipid-based emulsifier, improves structural compatibility and contributes to overall system stability [16]. In general, interfacial interactions in such systems are strongly influenced by surface-active components, and enhanced stability can be attributed to protein–lecithin synergism, in this case gelatin–lecithin interactions. Furthermore, lecithins can be physically and enzymatically modified to obtain emulsifiers with a broad range of hydrophilic–lipophilic balance values. Their role as stabilizing agents has been shown to combine the advantages of liposomal systems and polymer-based nanoparticles [48], while phospholipid surface modification improves biocompatibility.

Glycerine was incorporated as a plasticizer to enhance the flexibility and water retention capacity of the hydrogel network, thereby preserving mechanical integrity during extrusion and handling of printed structures [49]. Psyllium husk acted as a natural rheology modifier, contributing to viscosity and structural stability, while ascorbic acid functioned as an antioxidant, limiting oxidative degradation of lipid components [50,51]. Additionally, ascorbic acid may support the biological environment due to its role in cellular function and extracellular matrix synthesis.

Overall, the combination of protein-based (gelatin), lipid-based (fish oil), and polysaccharide-based (psyllium husk) components resulted in a hybrid hydrogel system with tunable rheological properties, sufficiently stable for printing, and potential bioactivity. The use of complementary emulsifiers ensured uniform dispersion of the lipid phase, while plasticizing and antioxidant agents further enhanced the functional stability of the system.

**Figure 8:**
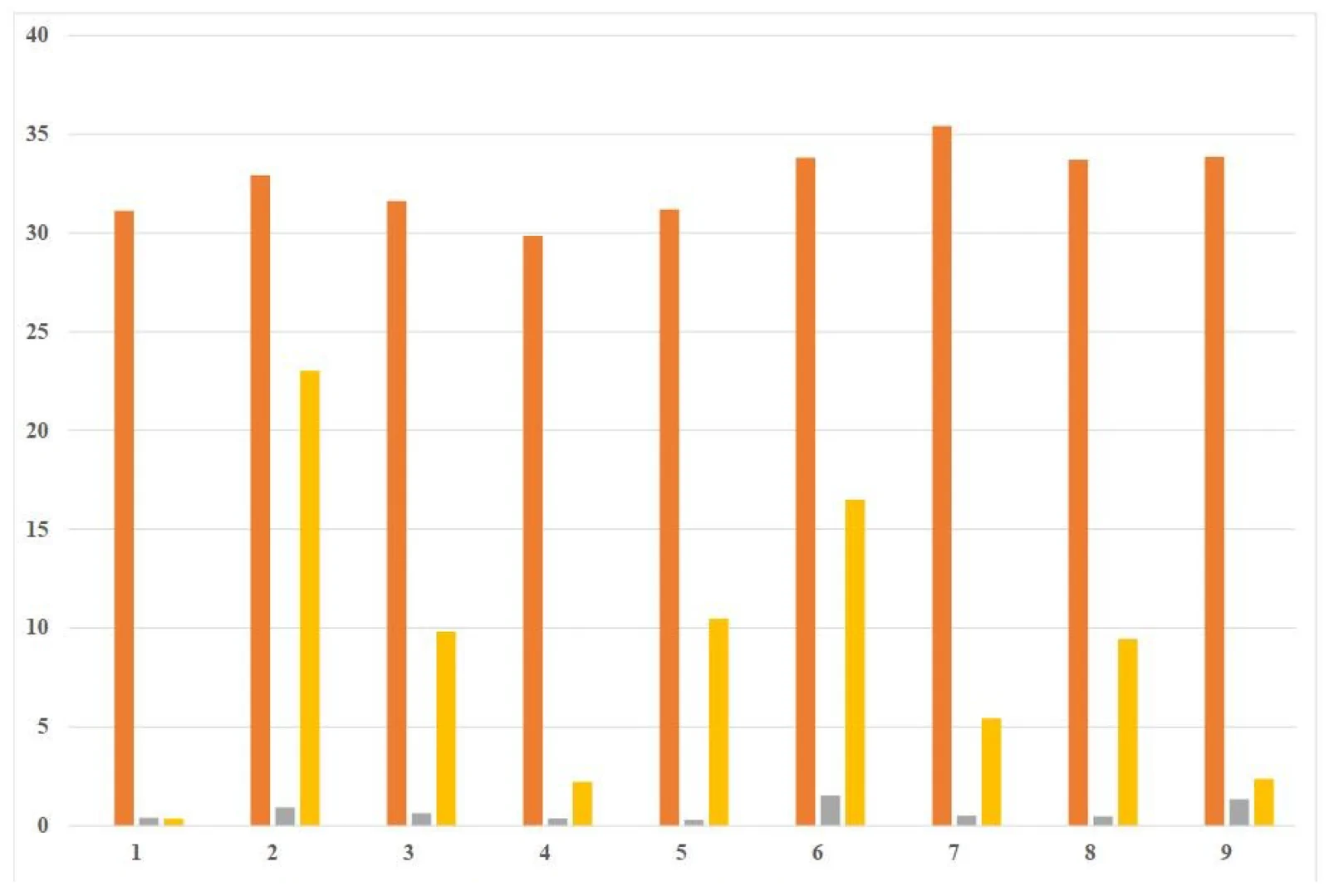
Mass changes [%] over consecutive storage periods for all samples. Samples are deonted at the x axis by 1–9 referring to corresponding S1–S9; Differences 0–3rd day [%] for each of the samples are denoted by orange color bars; Differences 3rd–7th day [%] for each of the samples are denoted by gray color bars; Differences 7th–110th day [%] for each of the samples are denoted by yellow color bars.

Mass loss was calculated after 3, 7, and 110 days, as shown in Table 6, from experimentally measured mass results, to evaluate the stability of hydrogel samples. Graphical representation of amounts of mass loss over time for all samples is shown in Figure 8.

The pronounced mass loss observed during storage, reaching up to approximately 91% after 110 days, was accompanied by progressive dehydration and a transition toward a more brittle state. This behavior represents an important limitation for applications requiring long-term structural integrity. Because the formulation was not chemically crosslinked, the measured mass loss may reflect the combined effects of water loss, dissolution, swelling/deswelling, erosion, and structural disintegration rather than degradation alone. The results therefore highlight the need to balance the high water affinity of natural polymer components with sufficient network stability, as excessive swelling or rapid desiccation can compromise the structural reliability of composite biomaterials [52]. Striking a balance between the inherently hydrophilic nature of the natural polymers used and the maintenance of structural stability is a known hurdle in hydrogel processing [53]. Although the samples retained a solid form after 110 days, this should not be interpreted as evidence of functional stability under physiological or application-specific conditions. Future optimization should therefore investigate crosslinking and other approaches for controlling water retention, swelling, mass loss, and mechanical integrity.

Density was experimentally measured 7 days after fabrication, and the results are presented in Table 7. The theoretically calculated density of the 3D printed samples (≈1.40 g/cm^3^, based on the formulation composition) differs significantly from the experimentally determined values. Similarly, the theoretically calculated sample masses (Table 5) differ substantially from the experimentally measured values. Given the importance of numerical modelling in the design of new material compositions, computational models should be provided with accurate input data. However, accurate measurement of the physical properties of hydrogels is challenging and requires careful consideration of their material characteristics.

Density is an intrinsic material property and, under identical conditions and for samples with the same composition and internal structure, should have essentially the same value. However, the densities reported in Table 7 range from 0.22 to 1.24 g/cm^3^, with an average value of 0.5 g/cm^3^. This is in line with the fact that most hydrated hydrogels reported in the literature exhibit bulk densities close to that of water (typically ~0.7–1.3 g/cm^3^), with the measured density depending strongly on polymer composition, water content, porosity, swelling state, and the measurement methodology [54]. It should be noted that sample S6 exhibited the highest value of 1.24 g/cm^3^ what could explain quite different mechanical behavior of this sample, as shown in the next chapters. On the other hand, several samples (S1, S3, S4, S7) had rather low densities. However, the present hydrogels have a novel composition, and lower densities may therefore be possible. Furthermore, the wide range of reported density values may suggest substantial differences in porosity, void content, or the degree of curing resulting from variations in the 3D printing parameters, although other factors, such as differences in composition or measurement uncertainty, may also contribute. It would be worthwhile to investigate how variations in input parameters, particularly physical properties such as density, affect the accuracy and reliability of currently used computational models.

**Figure 9:**
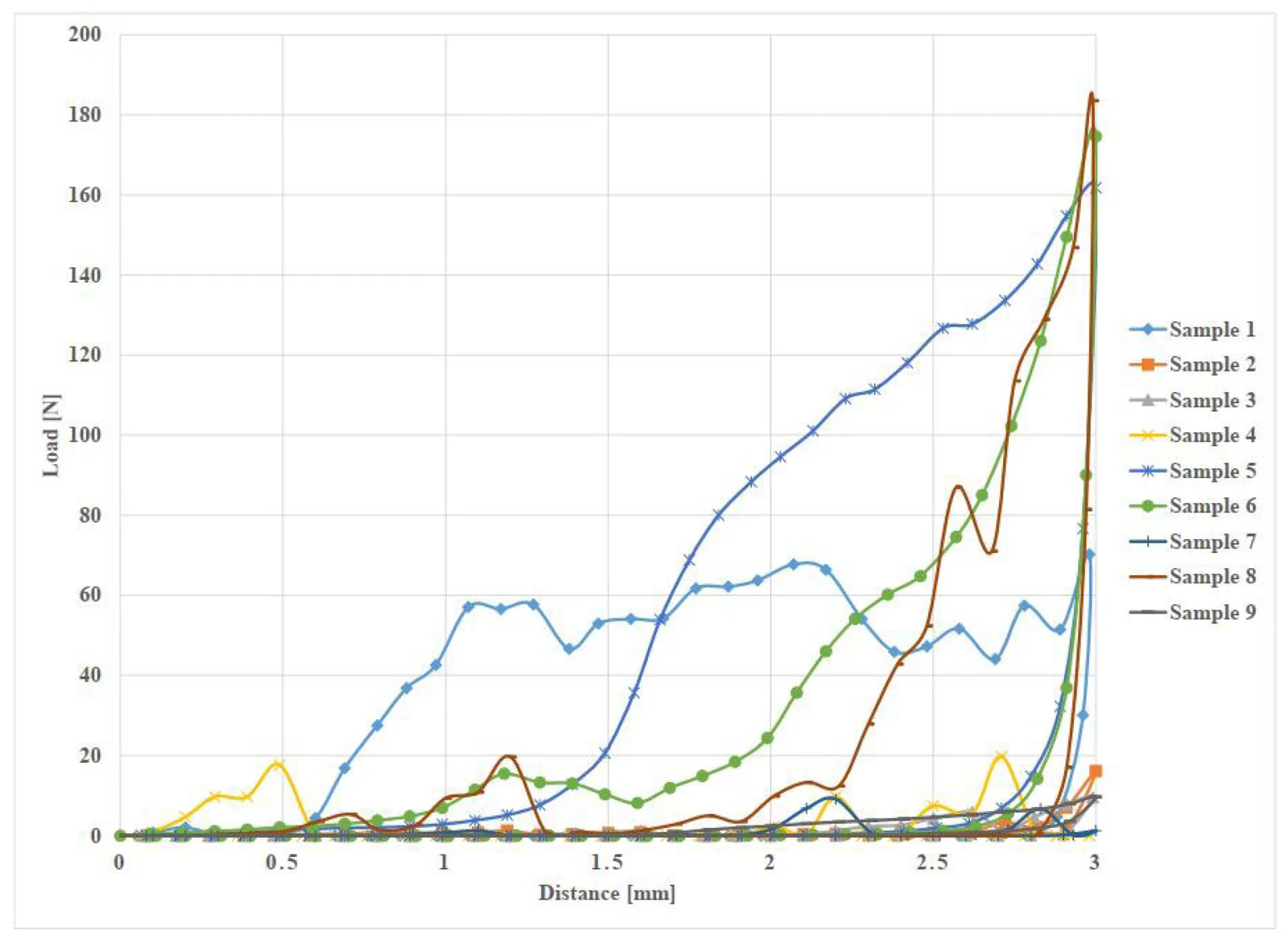
Load-displacement curves for flat indenter.

**Figure 10:**
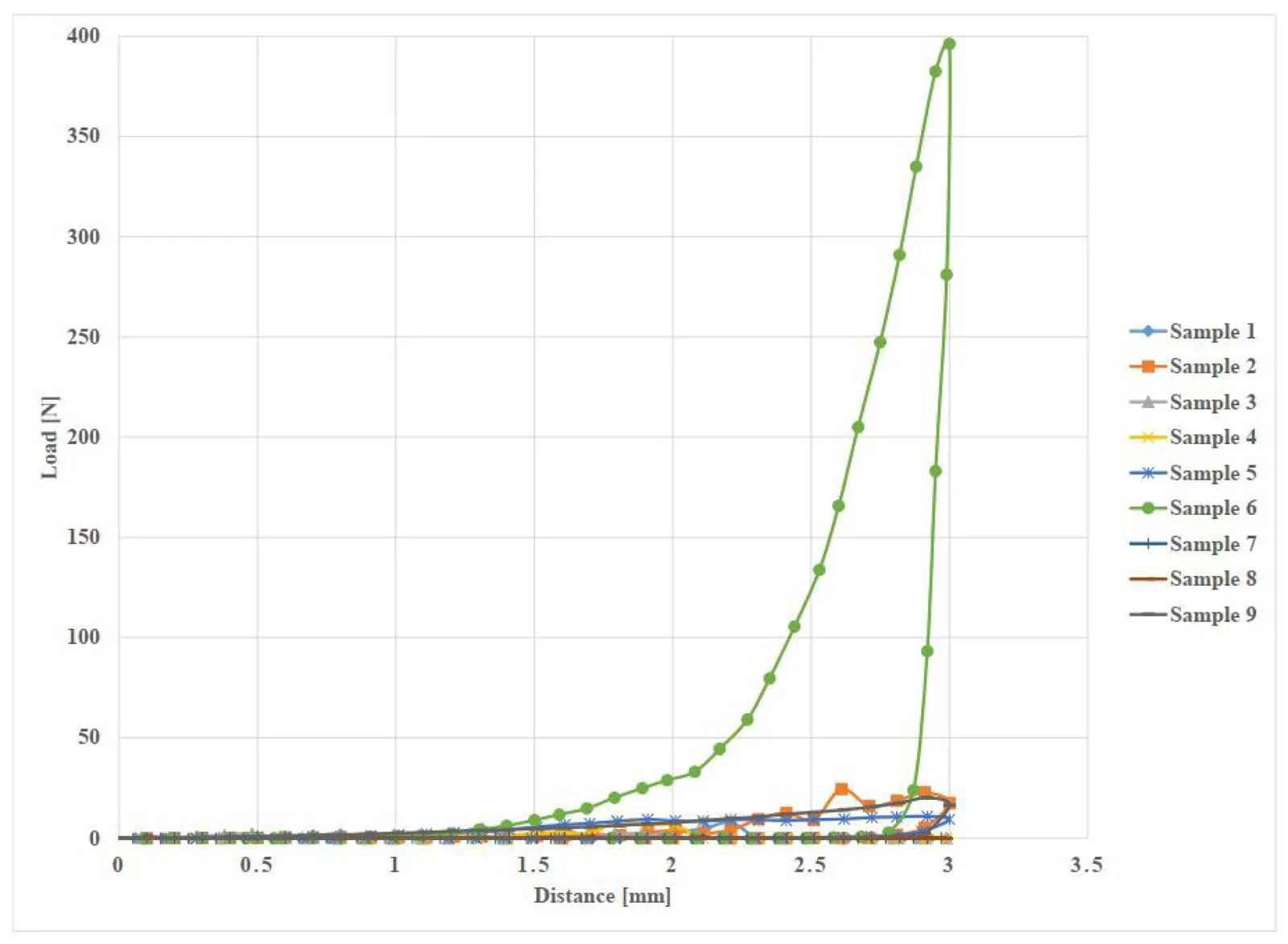
Load-displacement curves for spherical indenter.

For the analysis of mechanical responses, the geometry of the indenter needs to be considered. A flat-end cylindrical indenter is in contact with a relatively large part of the sample and therefore provides a more general measure of structural resistance. In contrast, spherical indentation generates a localized and nonuniform stress field and is more sensitive to local variations in composition, porosity, and filament arrangement. In this study, the flat indenter was used to evaluate the overall load-bearing behavior of the printed structures, whereas spherical indentation was used to examine the localized mechanical response and heterogeneity of the structure. Results of the compression tests with a flat cylindrical indenter are shown in Figure 9, and for a spherical indenter in Figure 10. The maximum compression loads achieved for each of the samples with a flat indenter and a spherical indenter are given in Table 8.

**Figure 11:**
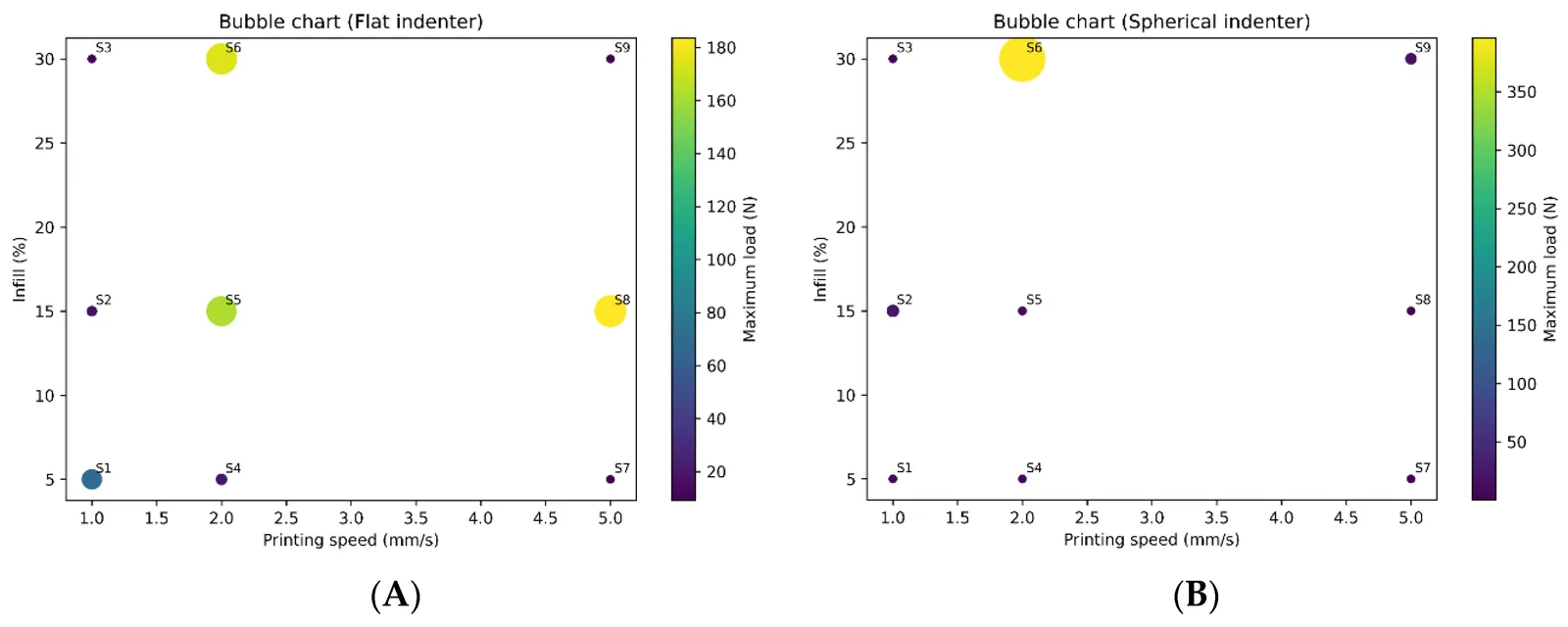
Bubble charts show the effect of printing speed and infill density on the maximum compression load of the 3D-printed gelatin-based formulations measured using (**A**) a flat indenter and (**B**) a spherical indenter. The *x*-axis corresponds to the printing speed (mm/s), the *y*-axis to the infill density (%), and both bubble size and color represent the measured maximum compression load (N); Sample IDs (S1–S9): shown next to each point.

**Figure 12:**
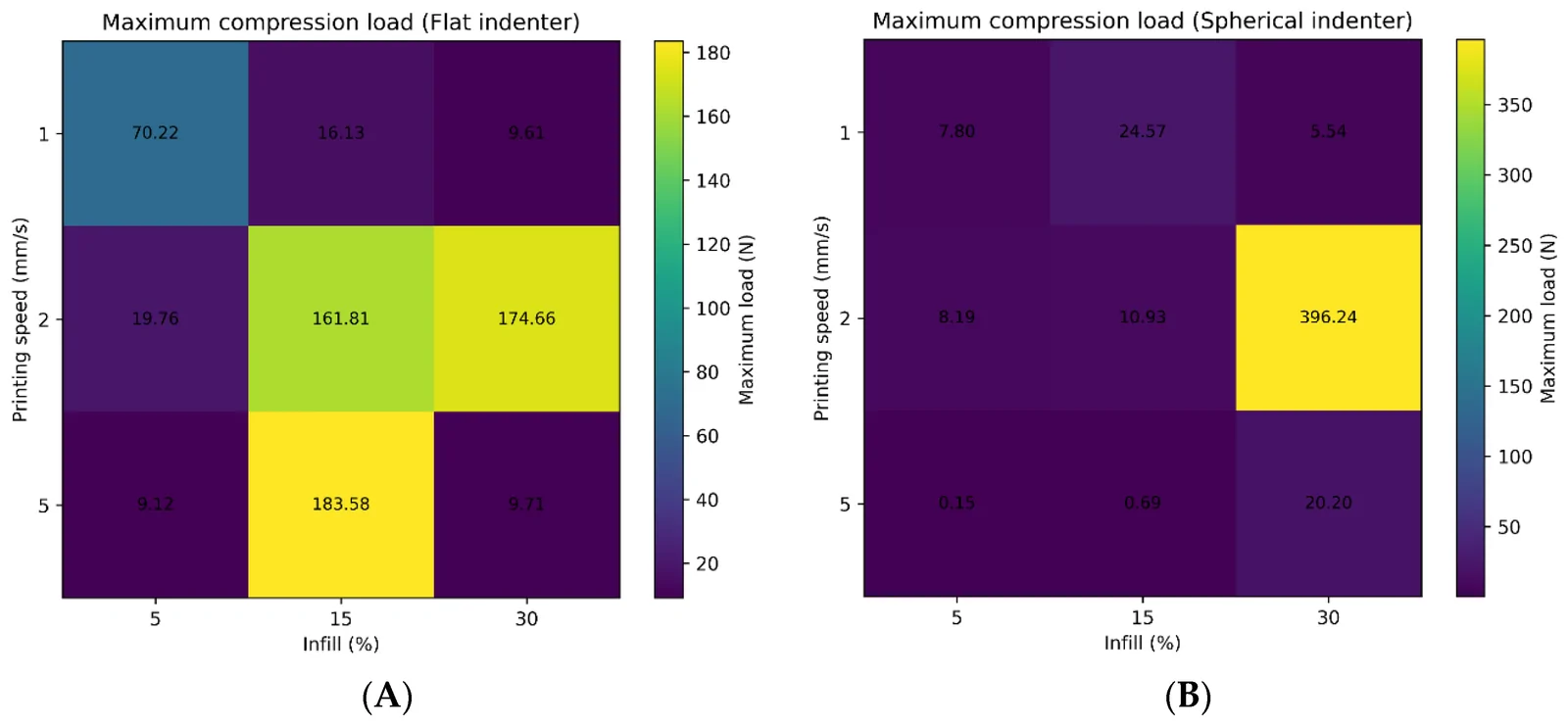
Heatmaps illustrating the influence of printing speed (1, 2, and 5 mm/s) and infill density (5, 15, and 30%) on the maximum compression load of 3D-printed gelatin-based formulations measured using (**A**) a flat indenter and (**B**) a spherical indenter. The numerical values within each cell represent the experimentally determined maximum compression load (N), while the color intensity reflects the relative mechanical performance of each printing condition, enabling direct comparison of the effects of fabrication parameters and indentation geometry on the load-bearing behavior of the printed constructs.

Effects of printing parameters on compression load are shown in bubble charts in Figure 11. Heatmaps illustrating the influence of printing speed and infill density on the maximum compression load are given in Figure 12. The plots facilitate visualization of the combined effects of fabrication parameters on the mechanical performance of the printed constructs and enable direct comparison between the two indentation methods.

It can be observed that the highest values of load for the flat indenter were measured with sample S8, followed by samples S6 and S5, respectively. Samples S5 and S6 were printed with a printing speed of 2 mm/s and S8 with 5 mm/s, thus indicating that an increase in printing speed should contribute to bearing load capacity. Samples S5 and S8 were fabricated with an infill value of 15%, while S6 had 30% infill. In general, comparison between these load values for samples with 5% infill and higher infill values clearly indicated that an increase in infill creates better load-bearing properties, which is expected. In all cases, maximum load was reached at approximately a maximum indentation depth of 3 mm, except for the sample S4 (at 2.71 mm indentation depth).

Compressive tests with a spherical indenter produced widely varying maximum loads, as expected. Sample S6 (30% infill and a printing speed of 2 mm/s) exhibited the highest compressive response (Figure 10, Figure 11B and Figure 12B), reaching a maximum load of 396.24 N at the prescribed indentation depth of 3 mm. S6 was the only sample to reach its maximum compressive load at the prescribed indentation depth. For all other samples, the maximum compressive load was reached before the spherical indenter attained the prescribed indentation depth of 3 mm, with sample S3 (30% infill and a printing speed of 1 mm/s) exhibiting the shallowest indentation depth (1.59 mm), at which the maximum load of 5.54 N was achieved. Continued displacement beyond the peak load did not produce additional load increase, indicating a reduction in the effective structural stiffness of the hydrogel. Such behavior is consistent with the poroviscoelastic nature of hydrogels, in which fluid exudation and stress relaxation reduce the load-bearing capacity during continued indentation. Furthermore, if local damage, crack initiation, or network collapse occurs beneath the spherical indenter, the load may decrease despite increasing indentation depth. Similar mechanisms have been reported for spherical indentation of hydrogels in the literature. It is generally attributed to damage, yielding, fracture, instability, or changes in contact conditions, rather than classical Hertzian elastic contact alone.

The exceptionally high maximum load measured for S6 (396.24 N) under spherical indentation indicates pronounced mechanical heterogeneity and represents an important limitation of the current formulation. Interestingly, S6 also exhibited the highest measured density (1.24 g/cm^3^), substantially exceeding the values obtained for the other samples. The simultaneous occurrence of increased density and maximum load suggests that differences in material compactness or local polymer content may have contributed to the distinct mechanical response of S6, However, this relationship should be interpreted as an association rather than direct evidence of causality. The observed variability may additionally arise from local differences in polymer distribution, porosity, network structure, hydration state, and indenter–sample contact conditions.

Future formulation development could therefore investigate reinforcement strategies to improve both mechanical strength and sample-to-sample uniformity. Cellulose nanocrystals (CNCs) and cellulose nanofibers (CNFs) are particularly promising candidates because their high aspect ratio, surface area, and ability to interact with polymer networks can reinforce hydrogel matrices while retaining favorable hydration characteristics and biocompatibility. Cellulose nanocrystals have been reported to enhance and modulate the mechanical properties of natural-polymer matrices [55]. This study on water-hyacinth-derived cellulose nanocrystals in PVA–chitosan films supports the potential of this general reinforcement strategy, although its applicability to gelatin bioinks remains to be experimentally established. Cellulose nanofiber incorporation has been experimentally shown to increase the mechanical properties of gelatin hydrogels while simultaneously modifying their rheological behavior and microstructure, with the effect depending on the reinforcement concentration [56]. More recent work using nanofibrillated cellulose in gelatin-based bioinks further demonstrated improvements in yield stress, compressive stress, and Young’s modulus, together with suitability for extrusion-based 3D bioprinting [57]. Gelatin/cellulose nanocrystal composite hydrogels have likewise demonstrated substantially enhanced mechanical strength while maintaining biocompatibility, particularly when nanocrystals were combined with enzymatic crosslinking [58]. Other reinforcement approaches, including nanoclay, silica-based nanoparticles, graphene-derived materials, and other inorganic or carbon-based nanofillers, could also be considered depending on the intended application [59,60]. However, their effects on cytocompatibility, rheology, printability, degradation, and long-term stability would require formulation-specific evaluation. Thus, future optimization should consider not only filler concentration and dispersion but also the interaction between reinforcement, polymer concentration, density, porosity, crosslinking, and printing parameters to achieve a more homogeneous mechanical response.

It is shown that spherical indentation of gelatin-based gels is governed by viscoelasticity and poroelasticity rather than purely elastic Hertzian contact [61]. They further demonstrated that reliable indentation measurements are obtained when the indentation depth is limited to approximately 15–25% of the spherical indenter radius [61]. For thin layers of hydrogels, load evolution during spherical indentation (at prescribed depths) exhibits poroelastic relaxation [62]. The force does not necessarily increase monotonically because fluid redistribution reduces the load after an initial peak. Spherical indentation curves of hydrogels exhibit significant viscoelastic creep and unloading effects, and loading is strongly rate-dependent [63]. During indentation, water is expelled from the contact region, and after an initial increase, the load may plateau or decrease even though indentation continues [62,64].

The maximum compression loads were generally higher when measured using the flat indenter than with the spherical indenter, except for sample S6 (Figure 9, Figure 10, Figure 11 and Figure 12). This difference can be attributed to the distinct contact geometries and stress distributions generated by the two indentation methods, which influence the measured mechanical response of soft hydrogel materials. Although increased infill density is generally expected to enhance the load-bearing capacity of 3D printed structures, the present results indicate that the mechanical response depends on a combination of factors, including indenter geometry, contact location, printing parameters, and hydrogel architecture.

In contrast, the effect of printing speed on the maximum load was less pronounced in our tests. Overall, the results suggest that the composition and internal network structure of the hydrogel exert the dominant influence on its complex compressive behavior. Additional studies covering broader ranges of printing parameters and contact conditions are required to further understand these interactions.

Contact geometry (flat vs. spherical) changes the measured force-displacement behavior because they generate different stress distributions and contact areas, producing different measured stiffness and force responses [65,66]. This distinction is particularly important for spherical indentation, where the initially small and evolving contact area makes the measurement more sensitive to local structural heterogeneity [67].

The different mechanical responses can also be interpreted in terms of the local contact mechanics and fluid transport pathways generated by the two indenter geometries. The flat cylindrical indenter produces a relatively large and approximately uniform contact area, resulting in a broader distribution of compressive stress and greater lateral confinement of the hydrogel beneath the indenter. This geometry can therefore probe the collective load-bearing response of a larger portion of the printed network while restricting local deformation and potentially limiting the escape of interstitial fluid from the highly compressed region. In contrast, the spherical indenter initially produces a much smaller contact area, with the contact radius increasing progressively with indentation depth. The resulting stress field is strongly localized beneath and around the spherical contact, generating pronounced stress gradients and greater sensitivity to local pores, filament interfaces, defects, and variations in network density. At the same time, the non-uniform contact geometry provides different pathways for fluid redistribution from the compressed zone toward surrounding less-confined regions. Consequently, poroelastic relaxation and fluid exudation can evolve differently under spherical and flat indentation, leading to substantially different load–displacement responses even for the same specimen. This effect is particularly relevant for S6, which exhibited an exceptionally high spherical-indentation load of 396.24 N at 3 mm displacement, whereas its response under flat indentation was 174.66 N. The markedly different response of S6 may indicate that the spherical probe encountered a locally dense or highly interconnected region of the printed network that was not representative of the larger volume sampled by the flat indenter. Its relatively high measured density (1.24 g/cm^3^) further supports the possibility of local differences in material compactness, although density alone cannot establish the cause of the anomalous mechanical response. Thus, the S6 result illustrates that spherical indentation may be particularly sensitive to local structural heterogeneity, while flat indentation provides a more spatially averaged response. These differences provide a mechanistic basis for the high variability in the stress ratio between the two methods and support the conclusion that the measurements should not be considered directly interchangeable.

To provide a quantitative interpretation of the different responses obtained with the two indentation geometries, the nominal contact area was considered in addition to the measured force. For the spherical indenter, with radius R = 12.7 mm, the Hertzian contact radius was estimated as a = (Rd)^1/2^, giving a depth-dependent contact area A_s_ = πRd, where d is the indentation depth at the maximum load. For the flat-indenter analysis, the reference cross-sectional area A_0_ = 900 mm^2^ used for the stress calculations was retained. The corresponding nominal contact pressures were estimated as p_s_ = F_s_/A_s_ and p_f_ = F_f_/A_0_. For S6, which reached 396.24 N at 3 mm spherical indentation, the estimated spherical contact radius and contact area were approximately 6.17 mm and 119.6 mm^2^, respectively. Thus, the spherical contact area was approximately 7.5 times smaller than the 900 mm^2^ reference area used for the flat-indenter calculation. Although the spherical load was only approximately 2.27 times higher than the flat-indenter load for S6, normalization by the estimated contact area resulted in substantially higher nominal contact pressure (approximately 3.31 MPa versus 0.194 MPa). This comparison illustrates that the exceptional S6 response cannot be interpreted solely from force values, because the two indenters interrogate markedly different spatial regions of the heterogeneous construct. The spherical probe concentrates the load within a relatively localized region and is therefore more sensitive to local variations in polymer density, porosity, filament arrangement and network structure, whereas the flat indenter provides a more spatially averaged response. From a poroelastic perspective, the different confinement geometries also generate different characteristic pathways for interstitial-fluid redistribution. The characteristic relaxation time can be expressed as t_p_ ∼ L^2^/D_p_, where L is the characteristic drainage distance and D_p_ is the effective poroelastic diffusivity. Although permeability and fluid-pressure measurements were not performed in the present study and therefore direct quantification of fluid exudation is not possible, this framework indicates that the two indentation geometries can produce different rates and spatial patterns of fluid redistribution and stress relaxation. These contact-area and poroelastic considerations provide a quantitative basis for interpreting the pronounced difference observed for S6 and support the conclusion that flat and spherical indentation measurements should not be considered directly interchangeable.

The force–displacement responses also indicate that the mechanical behavior of the printed hydrogels cannot be described as purely elastic. The spherical-indentation curves showed substantial variation in the indentation depth at which the maximum load was reached, ranging from 1.59 mm for S3 to 3.00 mm for S6, with several samples reaching their maximum load before the prescribed 3 mm displacement. In particular, after the peak load, continued indentation did not necessarily result in a further increase in force, which is consistent with a combination of time-dependent viscoelastic deformation, poroelastic fluid redistribution, and structural rearrangement under increasing deformation. Such behavior is consistent with previous indentation studies of hydrogels reporting viscoelastic and poroelastic contributions to the measured response [62,63,64]. The exceptionally different response of S6, which reached 396.24 N at 3 mm while the other samples reached substantially lower maximum loads at earlier or comparable depths, further demonstrates that the time-dependent response is strongly coupled with the local network structure and contact geometry. Because the present experiments did not include a displacement-hold or controlled loading-rate series, quantitative viscoelastic parameters such as relaxation time or equilibrium modulus could not be determined. Therefore, the present results should be interpreted as evidence of a time-dependent mechanical contribution rather than as a direct quantitative measurement of viscoelasticity.

The influence of printing parameters (printing speed and infill density) is highly complex, as discussed in a comprehensive review demonstrating that printability, extrusion parameters, filament architecture, infill density, and rheological properties jointly determine the mechanical performance of printed hydrogel constructs [68]. The review also emphasized that no single parameter independently determines the mechanical performance. A thorough understanding of rheology and its correlation with printability is essential for optimizing hydrogel printing and still challenging, especially for new hydrogel compositions. In general, chemically cross-linked hydrogels behave as pure solids with an elastic response, while physically cross-linked hydrogels exhibit viscoelastic properties [68]. Mechanical properties depend on composition, architecture, fabrication conditions, and crosslinking rather than on one variable alone [69].

The influence of contact position and contact area is significant, as demonstrated in a review showing that probe geometry, probe diameter, sample size, and contact area strongly affect measured compression values and should be considered when interpreting indentation experiments [70]. If the contact zone is small, as in the ideal case of a spherical indenter, various contact mechanics models can be applied [71]. However, all of these models have limitations in accurately determining the mechanical response because it depends on contact geometry and loading conditions that deviate from the ideal assumptions.

Above all other factors, hydrogel composition is the dominant factor governing its behavior under loading. Hydrogel mechanics are governed primarily by polymer chemistry, crosslink density, network structure, and water content rather than testing alone [72]. Mechanical behavior of hydrogels depends on chemistry, morphology, network configuration, viscoelasticity, and poroelasticity [73]. Determining the properties of a hydrogel is a complex task [72], even for seemingly straightforward measurements such as density. Many theoretical and constitutive hydrogel models assume a constant polymer density (or constant densities of the polymer and solvent) and derive the overall hydrogel density directly from the formulation composition and swelling state [74,75]. However, our experimental results demonstrate that density is a dynamic property influenced by processing parameters, storage time, and environmental conditions, which is in line with published research [29]. Hydrogels continuously respond to environmental stimuli (water content, temperature, pH, ionic strength), which alter their swelling behavior, dimensions, and physical properties [72]. Final properties of hydrogels depend on polymer composition, crosslinking strategy, processing route, network architecture, and water content, rather than only on theoretical formulation calculations [76]. In addition, the measurement technique itself contributes to the variability of the measured values. Different analytical techniques probe different structural scales and therefore can yield different measured values depending on the method employed [77]. Hydrogel properties measured after fabrication depend not only on material formulation but also on printing conditions, post-processing, and characterization methodology [68], including time-dependent dynamic changes of properties. Nonetheless, modeling of hydrogel behavior is a valuable tool to assist in various aspects of the optimal formulation design.

We used a very simple model for initial evaluation. Using the experimentally obtained parameters (maximum force, F, and corresponding displacement, d), engineering deformation ε_eng_ = d/h_0_ and engineering stress σ_eng_ = F/A_0_ (where A_0_ = 900 mm^2^ and h_0_ = 10 mm) were calculated for the flat indenter. True stress and true (logarithmic) strain were calculated by Equations (6) and (7) [78].

σ_true_ = σ_eng_ · (1 − ε_eng_) (6)

ε_true_ = −ln · (1 − ε_eng_) (7)

True (logarithmic) strain represents the accumulated deformation relative to the continuously changing specimen dimensions during compression, providing a more accurate measure of large deformations than engineering strain.

For the spherical indenter, representative stress and strain were calculated according to Tabor’s method using Hertz theory and using experimentally obtained parameters (maximum force, F, and corresponding displacement, d, for this indenter type). Contact radius a was calculated for each depth (displacement, d) and spherical radius R = 12.7 mm per Equation (8):

(8) $$a=\sqrt{2\cdot \mathrm{Rd}-d^{2}}$$

Contact pressure p_m_ can be represented by Equation (9):

(9) $$p_{m}=\frac{F}{\pi \cdot a^{2}}$$

Representative strain ε_r_ is given by Equation (10):

(10) $$\varepsilon_{r}=0.2\cdot \frac{a}{R}$$

And representative stress σ_r_ by Equation (11):

(11) $$\sigma_{r}=\frac{p_{m}}{\psi }$$

where ψ represents the constraint factor, with generally adopted value of ψ = 2.2 for soft hydrogels from indentation mechanics [79].

**Figure 13:**
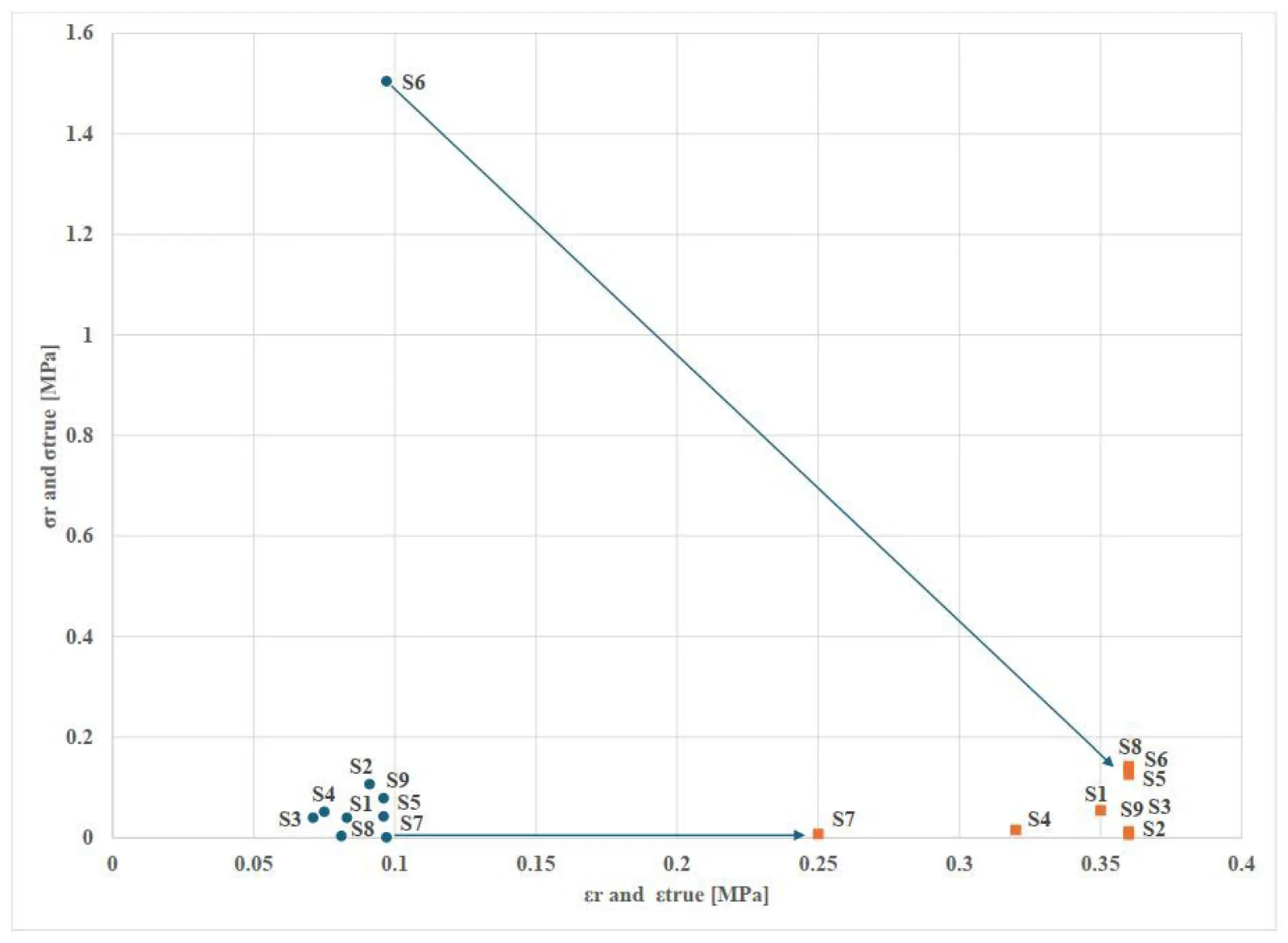
Paired stress–strain scatter plot for all samples (S1–S9): ε_r_—calculated representative strain with spherical indenter; σ_r_—calculated representative stress with spherical indenter; σ_true_—calculated true stress with flat indenter; ε*_true_*—calculated true (logarithmic) strain with flat indenter. Circle points represent spherical indentation, and square points represent flat indentation.

Results of these calculations are presented in Table 9 and as comparative scatter plots in Figure 13.

In Figure 13, each sample (S1–S9) appears twice: circle points for spherical indentation and square points for flat indentation. The two points belonging to the same sample are connected with an arrow for S6 and S7, as an example, to show the change in measured stress–strain coordinates when the testing method changes. Similarly, for all samples, the difference between the two points belonging to the same sample indicates the magnitude and shows the direction of change. It can be seen that the strain levels are significantly higher in the case of flat indentation, which was expected, while the magnitude of stress is comparable for the majority of samples. For sample S6, the stress level was significantly higher than in all other samples.

Using the calculated stress ratios presented in Table 9, the relationship between the stresses obtained by spherical indentation and flat compression can be summarized using three statistical parameters. First, the mean stress ratio was 4.43, calculated from the stress ratio column in Table 9, indicating that, on average, the representative stress obtained by spherical indentation was substantially higher than the corresponding true stress obtained by flat compression. Therefore, the observed mean value indicates a clear discrepancy between the measured stresses. Second, the standard deviation (SD) was 4.31, demonstrating considerable variation in the stress ratios among the investigated samples. Finally, the coefficient of variation (CV) reached 97.41%, indicating a very high relative variability and poor consistency of the stress ratio across the different formulations. These findings suggest that the relationship between spherical indentation and flat compression is not constant but is strongly influenced by the mechanical heterogeneity of the printed hydrogels, local contact conditions, and differences in stress distribution associated with the two loading geometries. Consequently, the stress obtained by one testing method cannot be directly predicted from the other using a single conversion factor, highlighting the need for method-specific mechanical characterization of these hydrogel formulations.

Furthermore, agreement between the flat and spherical indentation methods was evaluated using Bland–Altman analysis to show whether these two methods (spherical vs. flat compression) can be used interchangeably rather than whether they are correlated. For each sample, the following quantities were calculated: mean of the two methods, by Equation (12), and difference between methods, by Equation (13).

(12) $${\mathrm{Mean}}_{i}=\frac{\sigma_{r}+\sigma_{\mathrm{true}}}{2}$$

**Figure 14:**
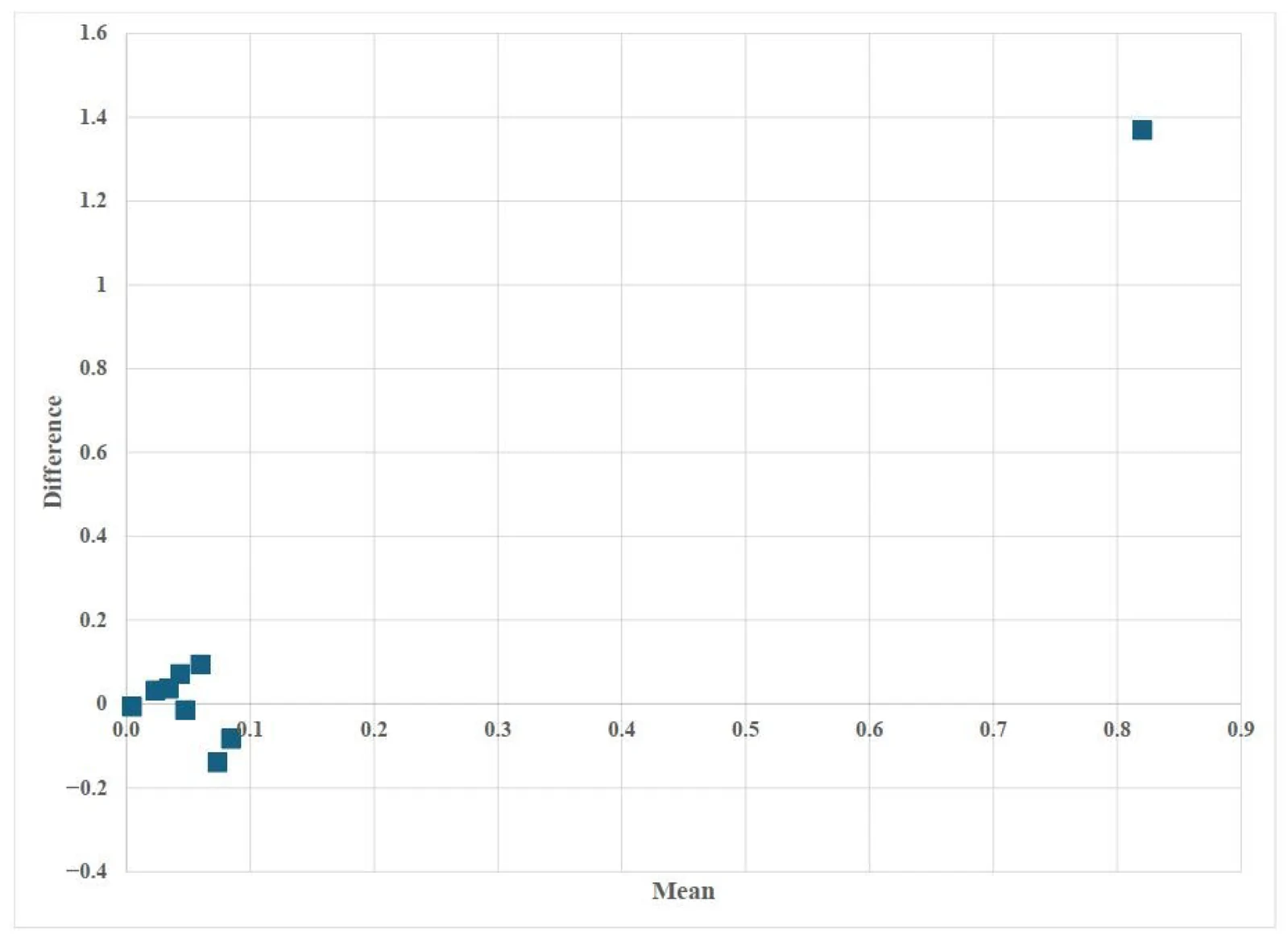
Bland–Altman plot.

Difference*_i_* = *σ_r_* − *σ_true_* (13)

where *σ_r_* is the stress obtained using the spherical indentation method, *σ_true_* stress obtained using the flat indentation method, and *i* represents the sample number. A positive value means the spherical indentation method measured higher than the flat method; a negative value means the spherical indentation method measured lower than the flat method, and a value of zero means the two methods agreed exactly for that sample. Having both positive and negative differences is completely normal and expected. In fact, if some differences are positive and others are negative, this often indicates no strong systematic bias between the methods. The Bland–Altman plot was constructed by plotting the difference between the two methods against their mean for each sample (Figure 14). This approach enables visualization of systematic bias and assessment of whether the agreement changes across the range of measurements. Mean bias was calculated by Equation (14), standard deviation of the differences, *SD_d_*, which quantifies the variability of the differences between the two methods, and 95% limits of agreement (LoA) by Equation (15).

(14) $$\bar{d}=\frac{1}{n}\sum_{i=1}^{n}(\sigma_{r}-\sigma_{\mathrm{true}})$$

where *n* is the total number of paired measurements (equals 9 for all samples here). The mean bias represents the average difference between the two methods and indicates whether one method systematically yields higher or lower values than the other.

(15) $$\bar{d}\pm 1.96\times SD_{d}$$

These limits define the interval within which approximately 95% of the differences between the flat and spherical indentation measurements are expected to lie, assuming the differences are approximately normally distributed. Accordingly, for spherical indentation and flat compression in our tests, mean bias is 0.15, standard deviation is 0.44, lower LoA is −0.70 and upper LoA is 1.01. Narrow limits of agreement indicate good agreement between the methods, whereas wider limits suggest greater variability and poorer interchangeability.

Agreement between the representative stress obtained from spherical indentation and the true stress obtained from flat compression was further evaluated using Bland–Altman analysis (Figure 14). The mean bias between the two methods was 0.15 MPa, indicating that, on average, spherical indentation yielded slightly higher stress values than flat compression. The standard deviation of the differences was 0.44 MPa, resulting in 95% limits of agreement ranging from −0.70 to 1.01 MPa. These relatively wide limits indicate substantial variability between the two measurement methods, suggesting that they should not be considered directly interchangeable. The Bland–Altman analysis complements the stress-ratio analysis by evaluating the absolute agreement between the two measurement methods rather than their proportional relationship. Although the mean bias is relatively small compared with the overall range of measured stresses, the wide limits of agreement demonstrate considerable variability among individual samples. This observation is consistent with the large coefficient of variation obtained for the stress ratios and indicates that the differences between spherical indentation and flat compression are sample dependent rather than constant. Such variability is expected for heterogeneous hydrogel materials, where differences in contact geometry, stress distribution, local microstructure, and viscoelastic response influence the measured mechanical behavior. Consequently, spherical indentation and flat compression should be regarded as complementary characterization techniques, each providing distinct information about the mechanical response of the printed hydrogel formulations.

The mean bias (0.15 MPa) appears relatively small, but one of the individual differences is approximately 1.37 MPa, which is much larger than the others. This suggests that the Bland–Altman analysis may be influenced by a single outlying sample (corresponding to previously identified S6, also represented by one far-right point in Figure 14). The relatively wide limits of agreement are influenced by one sample exhibiting a markedly larger difference between the two measurement methods, indicating that the agreement deteriorates for formulations with extreme mechanical responses.

The Bland–Altman plot also enables assessment of systematic bias, proportional trends across the measurement range, and potential outliers. In the present study, most samples formed a compact cluster at lower mean stress values, indicating relatively consistent agreement between the two measurement methods within this range. In contrast, sample S6 was clearly separated from the remaining samples along both the horizontal and vertical axis because of its substantially higher average stress. This observation indicates that S6 exhibited a markedly different mechanical response compared with the other formulations. Although the difference between the two methods remained within the calculated 95% limits of agreement, the isolated position of S6 suggests that the agreement between spherical indentation and flat compression may become more variable for samples with exceptionally high mechanical strength. This finding is consistent with the heterogeneous mechanical behavior observed in the stress-ratio analysis and highlights the influence of material composition and local contact mechanics on the measured response.

Water contact angle measurements showed that all investigated samples were hydrophilic, with θavg values ranging from 30.07° to 50.63° (Table 10). The lowest mean contact angle was recorded for S5 (30.07°; 2 mm/s, 15% infill), indicating the greatest relative wettability among the tested samples, whereas S3 showed the highest value (50.63°; 1 mm/s, 30% infill) and therefore the lowest relative wettability. Samples S4, S2, and S1 also exhibited comparatively low mean contact angles (31.72°, 33.33°, and 33.88°, respectively), while S6 and S9 were among the samples with higher contact angles (48.18° and 44.38°, respectively).

When the results were grouped according to printing parameters, infill percentage showed a more pronounced descriptive association with contact angle than printing speed (Table 11). Mean θavg values were 34.69°, 33.76°, and 47.73° for 5%, 15%, and 30% infill, respectively. In contrast, the means grouped by printing speed were 39.28°, 36.66°, and 40.24° for 1, 2, and 5 mm/s, respectively. The range between the level means was therefore 13.97° for infill and 3.59° for printing speed. Notably, the 30% infill condition yielded the highest contact angle at each of the three printing speeds, whereas the effect of printing speed was non-monotonic.

Based on the conventional water contact angle criterion, surfaces with θ < 90° are classified as hydrophilic, whereas surfaces with θ > 90° are classified as hydrophobic. Accordingly, all investigated samples were hydrophilic. Their relative wettability was ranked, where a lower θavg indicates higher wettability, with results presented in Table 12.

All nine samples exhibited mean water contact angles below 90°, confirming an overall hydrophilic surface behavior. This response is consistent with the water-interacting character of the final formulation, which contains an agar-based gelling blend, psyllium husk, glycerin, and the nonionic surfactant Tween 80. Naturally derived hydrogel networks can exhibit substantial water sorption and diffusion due to hydrophilic functional groups and interconnected polymer structures [80]. Although the formulation also contains omega-3 fish oil and soy lecithin, the measured contact angles indicate that the overall surface response remained hydrophilic.

Because all samples were prepared from the same final formulation and printed using the same line infill pattern, the differences observed among S1-S9 are more plausibly associated with the architecture produced by the printing parameters than with differences in chemical composition. Among the two investigated parameters, infill percentage showed the stronger descriptive association with wettability. The samples printed with 30% infill consistently exhibited higher contact angles at all three printing speeds (S3: 50.63°, S6: 48.18°, and S9: 44.38°) than the corresponding 5% and 15% infill samples. The marginal mean for 30% infill was 47.73°, compared with 34.69° and 33.76° for 5% and 15% infill, respectively.

Possible explanation is that changing infill modifies the fraction of deposited material, pore geometry, open surface area, and local topography, which can alter both droplet spreading and penetration into a porous scaffold. In 3D printed scaffolds, infill density has also been reported to influence water contact angle through changes in pore size and liquid penetration, and the relationship does not necessarily follow a simple linear trend [81]. Therefore, the higher contact angles observed at 30% infill may reflect a denser surface architecture that reduced rapid penetration or spreading of the water droplet in comparison to the more open 5% and 15% structures.

The interpretation of contact-angle data on these samples should also account for their rough and porous surface morphology. Classical wetting models demonstrate that surface roughness and the fraction of solid-liquid contact can substantially modify the apparent contact angle measured on a real surface [82,83]. Accordingly, the values obtained here should be interpreted as apparent wettability, reflecting the combined effects of surface chemistry, roughness, porosity, and liquid penetration rather than intrinsic surface chemistry alone.

Printing speed showed a smaller and non-monotonic descriptive effect. Samples printed at 2 mm/s had the lowest grouped mean contact angle (36.66°), compared with 39.28° at 1 mm/s and 40.24° at 5 mm/s. Printing speed may affect strand deposition, surface continuity, interlayer fusion, and local roughness. It would be beneficial to perform more comprehensive tests on wider range of surface-morphologies and roughness to better understand specific governing mechanism. Within the present experimental setting, infill percentage therefore appears to be the more influential printing parameter for apparent wettability, while printing speed seems to play a secondary role.

These findings should be interpreted as descriptive trends rather than statistically established main effects because each speed-infill combination was represented by a single printed scaffold. The three measurements per scaffold characterize spatial variation across the same specimen but are not independent printing replicates. In addition, water can be rapidly absorbed by porous hydrogel structures, causing the contact angle to change with time after droplet deposition [80]. Future measurements would therefore benefit from multiple independently printed samples per condition, a fixed image-acquisition time after droplet deposition, and automated contact-angle analysis. Nevertheless, the consistent increase in contact angles for the 30% infill condition across all three printing speeds supports the conclusion that scaffold architecture contributed substantially to the observed differences in wettability.

**Figure 15:**
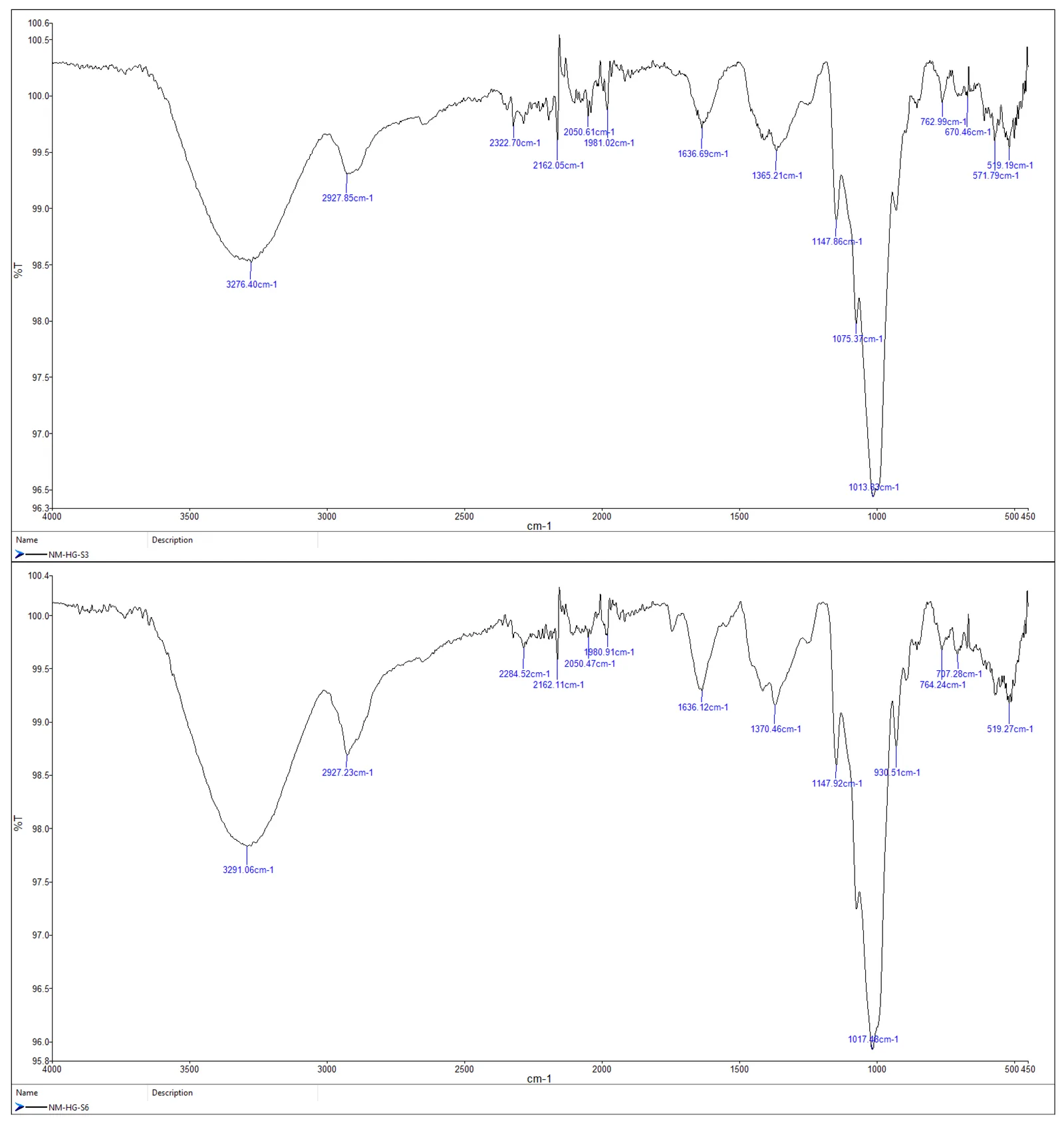
FTIR spectroscopy for samples S3 (**upper image**) and S6 (**lower image**).

FTIR spectroscopy was performed on all 3D printed samples with an identical material composition and different infill percentages in order to assess whether changes in the internal structure and infill density were associated with detectable changes in the chemical composition of the printed material. The spectra showed a similar overall profile across all samples, as shown in Figure 15, including samples S3 and S6, which exhibited markedly different mechanical behavior according to the data presented in Table 9. Among the samples with 30% infill, S3 exhibited the lowest maximum load, whereas S6 exhibited the highest maximum load, for both the spherical and flat indenters.

The results suggest that increasing the infill percentage did not lead to noticeable changes in the material’s chemical composition. A broad absorption band appeared in the 3276–3305 cm^−1^ range. This is mostly due to O–H stretching vibrations linked to water, glycerol, and polysaccharide/hydrocolloid parts of the matrix. Depending on the type of plant-based gelatin, N–H stretching vibrations might also play a role. The bands found at 2922–2929 cm^−1^ are mainly from aliphatic C–H stretching vibrations from glycerol, fish oil, Tween 80, lecithin, and polysaccharide components. The absorption bands in the 1634–1643 cm^−1^ region are mostly from deformation vibrations of bound water, with possible contributions from carbonyl/carboxylate groups and amide-I vibrations of protein components.

The bands seen at 1412–1417 cm^−1^ relate to symmetric stretching vibrations of COO^−^ groups and/or CH_2_ bending vibrations. The bands at 1365–1371 cm^−1^ mainly come from C–H/CH_3_ deformation vibrations. The absorption band around 1148 cm^−1^ can link to C–O–C stretching vibrations, including those from glycosidic linkages. The bands at 1075–1077 cm^−1^ match C–O stretching vibrations from polysaccharides and glycerol, with potential contributions from phosphate groups linked to lecithin. The region between 994 and 1017 cm^−1^, one of the most intense areas in the spectra, mainly relates to C–O and C–O–C vibrations of the polysaccharide-rich matrix. Additional bands at around 929–931 and 846–855 cm^−1^ can be linked to vibrations from saccharide ring structures and glycosidic linkages.

The lower-wavenumber regions at 760–706 and 573–520 cm^−1^ correspond to skeletal and other matrix-related vibrations, which are too general to assign to specific components.

An ester carbonyl absorption band, usually seen in the 1735–1750 cm^−1^ range due to fish oil, Tween 80, and lecithin, was not clearly identified in the spectra. This might be due to the relatively low concentration of these components or their overlap with stronger absorption bands from the main matrix components. Therefore, the absence of a clearly defined carbonyl peak should not be taken as proof that these constituents are missing. Notably, no clear increase or decrease in absorption intensity was seen with increasing infill percentage. Raising the infill from 5% to 30% did not cause new absorption bands to appear, the disappearance of key bands, consistent shifts in main absorption maxima, or other spectral indicators of new chemical bonds or material degradation. The observed changes in band intensity are likely tied to differences in the physical features of the 3D-printed samples, such as thickness, surface roughness, porosity and other influences.

For future studies, the mechanical performance of the material could be further enhanced through a crosslinking process, focusing on a nature-derived crosslinking agents, such as genipin crosslinking, which could improve the structural stability and mechanical integrity of the gelatin-based matrix [57,84,85].

Sample S6 (30% infill and a printing speed of 2 mm/s) exhibited the highest compressive response, with a higher maximum load under spherical than flat indentation, which was not expected. S6 also exhibited the highest density (1.24 g/cm^3^), approximately twice that of S5 and S8, the next densest samples, and more than five times that of S1 and S7. This substantially higher density may have contributed to the unusually high compressive response observed for S6. The 30% infill likely contributed to the mechanical behavior by increasing the effective material content, interfilament contact, and load-bearing capacity compared with samples printed at lower infill percentages. However, the results indicate that the mechanical response was not governed by infill percentage alone, but also depended on printing speed, indenter geometry, local contact conditions, and structural heterogeneity within the printed hydrogel. In particular, the combination of 30% infill and a printing speed of 2 mm/s may have resulted in greater dimensional inaccuracies or local over-deposition during printing, potentially explaining the substantially higher density of S6 compared with the other samples.

The exceptionally high response of S6 under spherical indentation was not accompanied by substantial differences in its FTIR spectrum, suggesting that its distinctive mechanical behavior was primarily associated with the printed architecture and local structural organization rather than changes in chemical composition.

For porous 3D printed structures, the measured compression response reflects both the intrinsic behavior of the material and the material structure produced during printing. Infill density changes the effective load-bearing area, filament spacing, and connectivity of the deposited material, whereas printing speed commonly influences filament continuity, dimensional accuracy, and interlayer contact. Consequently, samples produced from an identical material composition may exhibit substantially different load–displacement responses when their printing parameters are changed. Furthermore, composition differences may arise from variations in the 3D printing parameters if phase separation or material degradation occurs due to temperature effects. Collectively, these factors make the characterization of 3D-printed hydrogels a highly complex process.

The comprehensive characterization performed in the present study is consistent with the current international trend toward standardizing hydrogel evaluation for biomedical and biofabrication applications. In particular, the combination of storage stability assessment, density measurements, optical microscopy, and complementary compression testing using flat and spherical indenters addresses several areas currently recognized as priorities by ISO/TC 276 (ISO/WD TS 25812) and ASTM Committee F04.42 [25]. These ongoing initiatives aim to establish harmonized procedures for evaluating hydrogel functionality, mechanical performance, quality control, and printability, while identifying remaining gaps in standardized methods for bioinks and 3D printed hydrogels. The observed sensitivity of the measured properties to formulation, processing parameters, storage conditions, and testing geometry further highlights the importance of developing robust and reproducible characterization protocols that enable meaningful comparison of results between laboratories. Consequently, the methodology presented in this study contributes to the broader effort toward establishing reliable performance evaluation frameworks for extrusion-printed hydrogel systems [28].

### 4.1. Limitations and Future Perspectives

The characterization of the present gelatin-based hydrogel remains challenging because printability, rheology, emulsion stability, and mechanical performance are strongly interdependent [2,68]. The incorporation of fish oil creates a dispersed lipid phase that must remain uniformly distributed during mixing, extrusion, and storage, while Tween 80 and lecithin are required to stabilize the interface and prevent phase separation [16,44,45]. At the same time, psyllium husk modifies viscosity and network stability [14], whereas glycerin improves flexibility and water retention [48]. These interacting effects make optimization of a single formulation difficult because changes introduced to improve extrusion behavior can simultaneously alter porosity, dehydration rate, and compressive response. In addition, extrusion-based bioprinting is highly sensitive to printing parameters, and the final properties of printed hydrogels depend not only on composition but also on filament architecture, infill density, interlayer bonding, and post-processing conditions [2,5,66]. The large variability observed in density and indentation-based stress measurements further indicates that local heterogeneity and contact geometry can strongly influence the measured response [50,61,62,68]. This observation is consistent with the substantial variability observed among the printed samples, particularly in density and stress-ratio analysis.

The desired rheological properties can be achieved by optimizing the formulation to ensure a stable and homogeneous dispersion of all components before and during printing. In the present system, phase separation can be minimized through appropriate mixing and homogenization, control of component ratios, and optimization of the viscosity and gelation behavior of the gelatin–based matrix. The FTIR results showed no evidence of substantial differences in chemical composition among samples printed using different speeds and infill densities, while the observed mechanical variations were mainly attributed to differences in the printed architecture and local structural heterogeneity. Nevertheless, phase stability remains an important consideration, particularly for formulations containing immiscible or particulate components, and should be further evaluated through rheological measurements, microscopy, and time-dependent stability studies.

The interpretation of the mechanical response was additionally complicated by the strong influence of the testing conditions. Contact geometry, indenter position, contact area, sample architecture, and local structural variations all affected the measured load–displacement behavior. In particular, flat and spherical indenters probed different aspects of the material response and generated different stress distributions within the samples. Therefore, the results obtained using these methods could not be directly compared or converted using a single universal relationship. Another limitation was the use of simplified mechanical models, which provided only an initial approximation of the complex behavior of the printed hydrogel. Such models do not fully account for viscoelasticity, poroelasticity, fluid redistribution, local damage, structural collapse, or the heterogeneous nature of the printed network. In addition, the relatively small number of samples and the limited range of printing speeds and infill densities reduced the possibility of establishing robust and statistically supported relationships between processing parameters and mechanical performance.

The maintenance of porosity in the proposed bioink would depend on its intended application and the required balance between mechanical integrity, mass transport, and cellular infiltration. In extrusion-based bioprinting, the macroporous architecture can be controlled through the printing design, including filament spacing, layer arrangement, infill percentage, and pore geometry. However, the final porosity may also be affected by swelling, crosslinking, degradation, and the surrounding environment. Therefore, for future biomedical applications, the printing and post-processing conditions should be optimized according to the specific application to ensure that the desired porous structure is maintained while providing sufficient mechanical stability. In the present study, the investigated infill densities demonstrate that printing architecture can substantially influence the physical and mechanical properties of the constructs. However, detailed evaluation of pore stability and biological performance remains necessary before defining a specific biomedical application.

The absence of a crosslinking step represents an important limitation of the present formulation. Therefore, the observed loss of material over time may involve dissolution, swelling, erosion, and structural disintegration rather than degradation alone. Although the samples retained a solid form after 110 days under the investigated storage conditions, this observation does not demonstrate their long-term stability under physiological or application-specific conditions. Future studies should therefore investigate appropriate crosslinking strategies, such as genipin-mediated crosslinking of the gelatin-based matrix, which has been shown to increase the mechanical stiffness and structural stability of gelatin materials while reducing swelling and improving resistance to dissolution [57,84,85]. The effects of crosslinking on printability, swelling, mass loss, mechanical properties, and application-specific performance should be systematically evaluated. For topical applications such as cosmetic patches, the required stability period would depend on the intended duration of use, whereas a controlled dissolution or disintegration profile could potentially be advantageous for the release of incorporated active compounds.

Consequently, the present study should be regarded as a preliminary but comprehensive mechanical characterization of a novel gelatin–fish oil hydrogel system, and future work should include rheological characterization, controlled humidity storage, microstructural imaging, and biological evaluation to establish the relationship between formulation, printing conditions, and long-term functional performance [23,66]. Future investigations should include a larger number of samples, a broader range of printing and storage conditions, more accurate determination of density, porosity, and water content, as well as detailed rheological and microstructural analyses. The development of more advanced mechanical and computational models, together with biological evaluation, would provide a more comprehensive understanding of the material and support the assessment of its suitability for specific biomedical and bioprinting applications.

### 4.2. Biocompatibility Considerations and Potential Application as a Cosmetic Patch

The components used in the investigated formulation were selected primarily based on their expected functional contributions to gel formation, printability, emulsion stabilization, flexibility, or potential cosmetic activity. The formulation was designed using predominantly biocompatible and naturally derived or bio-based components, selected for their potential functional roles in hydrogel formation, hydration, lipid-phase stabilization, and topical application. However, the biocompatibility and sustainability of the final multicomponent formulation depend on the concentration, purity, source, manufacturing route, and interactions among individual constituents and therefore require application-specific experimental and life-cycle evaluation.

The formulation contained an agar-based gelling blend, omega-3 fish oil, polysorbate 80, soy lecithin, glycerin, psyllium husk powder, ethanol, L-ascorbic acid, hyaluronic acid, and demineralized water. Several of these components, particularly natural polysaccharides, hyaluronic acid, lipid-based ingredients, and pharmaceutical excipients, have established applications in food, cosmetic, pharmaceutical, topical, or biomedical formulations [86,87,88,89]. Nevertheless, the safety and biocompatibility of the final multicomponent formulation cannot be inferred solely from the individual use history of its constituents and require application-specific experimental evaluation. Also, the presence of individually biocompatible components does not automatically demonstrate the biocompatibility of the final multicomponent formulation.

The agar-based gelling component provides structural integrity [86], while hyaluronic acid is widely used in topical and biomedical formulations because of its hydration-related properties and general compatibility with biological environments [87]. Glycerin is commonly used as a humectant and plasticizing agent, whereas soy lecithin can contribute to emulsification and stabilization of lipid-containing formulations [90]. Omega-3 fatty acids and L-ascorbic acid may provide functional benefits relevant to cosmetic applications, including support for skin barrier function and modulation of skin inflammation in the case of omega-3 fatty acids, and antioxidant activity and support of collagen biosynthesis in the case of L-ascorbic acid [88,91,92]. Psyllium husk is a polysaccharide-rich material with high water-uptake and gelling capacity that can contribute to water retention and modify the rheological behavior of hydrogel formulations [93]. Nevertheless, the biological response to these components may depend on their concentration, source, molecular characteristics, purity, and interactions with other formulation constituents.

Particular attention is required for polysorbate 80 and ethanol. Polysorbate 80 is widely used as a nonionic surfactant, emulsifier, and formulation excipient. However, its biological effects can depend on formulation composition, concentration, and exposure conditions, supporting the need to optimize its concentration and experimentally evaluate the compatibility of the final formulation, particularly for prolonged topical application [94,95]. The Cosmetic Ingredient Review (CIR) Expert Panel has also evaluated polysorbates as used in cosmetics and concluded that they can be considered safe when formulated to be non-irritating [96]. Likewise, ethanol was used as part of the preparation procedure. However, residual ethanol in the final construct may affect cell viability and skin-barrier function depending on its concentration and exposure duration. Consequently, the ethanol content and potential evaporation during preparation and printing should be controlled in any formulation intended for direct biological or topical use [97,98,99].

For a potential cosmetic patch application, the requirements differ from those of an implantable scaffold. The formulation would primarily require compatibility with the skin, adequate physical stability, maintenance of the desired hydration and mechanical properties, and the absence of unacceptable irritation or sensitization. In contrast, claims regarding scaffold biocompatibility, cellular proliferation, tissue integration, or long-term implantation cannot be made based solely on the present physicochemical and mechanical characterization. Before practical application, the final optimized formulation would therefore require systematic evaluation of cytocompatibility, skin irritation and sensitization potential, microbiological quality, formulation stability, and the influence of component grade and purity.

Accordingly, the present study should be considered a preliminary investigation of the formulation and its processing and mechanical characteristics rather than a demonstration of biological safety or clinical suitability. Further biological evaluation of the final optimized formulation will be necessary before its use as a cosmetic patch or adaptation for biomedical applications.

## 5. Conclusions

A novel multicomponent gelatin-based hydrogel containing fish oil, Tween 80, lecithin, glycerin, psyllium husk, and ascorbic acid was initially designed using an AI-supported formulation approach and subsequently refined through experimental optimization for extrusion-based 3D printing. Nine acellular constructs were fabricated at printing speeds of 1–5 mm/s and infill densities of 5–30%. The formulation demonstrated sufficient consistency for extrusion and fabrication of constructs with different printed architectures, although substantial differences in physical and mechanical properties were observed among samples.

Storage testing revealed pronounced dehydration, with mass losses exceeding 60% after 7 days and reaching up to approximately 91% after 110 days under the investigated dry-air conditions. Experimentally measured densities ranged from 0.22 to 1.24 g/cm^3^ and differed substantially from the theoretically calculated density, demonstrating that density cannot be assumed solely from formulation composition and may be strongly affected by printed architecture, porosity, storage conditions, and measurement methodology. Sample S6 exhibited the highest measured density (1.24 g/cm^3^), which may have contributed to its substantially different mechanical response.

Wettability measurements showed that all samples had hydrophilic surfaces, with apparent water contact angles between 30.07° and 50.63°. The descriptive results indicated a stronger influence of infill density than printing speed on apparent wettability, with the 30% infill samples consistently exhibiting higher contact angles. Because the investigated surfaces were porous and rough, these measurements represent apparent rather than intrinsic wettability and reflect the combined effects of surface chemistry, porosity, topography, and water penetration.

ATR-FTIR analysis of all nine samples showed similar overall spectral profiles despite substantial differences in printing parameters and mechanical behavior. For example, the spectra of S3 and S6 were broadly similar despite their markedly different compressive responses. No clear spectral evidence of new chemical bonds or substantial chemical changes associated with increasing infill density was identified. These results indicate that the major differences in mechanical behavior were more likely associated with physical architecture, density, porosity, and local structural organization than with detectable changes in chemical composition.

Compression testing demonstrated that flat cylindrical and spherical indentation provide complementary rather than interchangeable information. Flat indentation generally produced higher maximum loads and provided a more spatially averaged response, whereas spherical indentation generated a more localized contact condition and was consequently more sensitive to local heterogeneity. Sample S6 represented a pronounced exception, reaching 396.24 N under spherical indentation and exhibiting substantially different behavior from the other samples. Its high density, together with the relatively small spherical contact area, provides a possible explanation for its sensitivity to local compactness and structural organization. The contact-area analysis therefore demonstrates that differences in measured force cannot be interpreted independently of indentation geometry.

Stress-ratio analysis and Bland–Altman evaluation showed that the two testing methods cannot be considered directly interchangeable. The mean stress ratio (σr/σtrue = 4.43) and the high coefficient of variation (97.41%) indicated substantial variability among samples, while the Bland–Altman limits of agreement (−0.70 to 1.01 MPa) demonstrated considerable sample-dependent differences between spherical and flat compression. These results demonstrate that spherical and flat indentation should not be considered directly interchangeable for the investigated heterogeneous hydrogel constructs.

The AI-supported formulation approach was useful for generating an initial multicomponent formulation concept, while the present results demonstrate that experimental optimization and comprehensive characterization remain essential for establishing the suitability and performance of complex hydrogel formulations.

Overall, the results demonstrate that the behavior of the printed hydrogel is controlled by the combined effects of formulation, physical network organization, density, porosity, storage state, printing architecture, and contact geometry. The combination of optical characterization, storage stability, density, wettability, FTIR, and complementary mechanical testing provides a more comprehensive assessment than printability or a single mechanical test alone. Nevertheless, the absence of chemical crosslinking, limited number of independently printed samples, restricted printing-parameter range, and lack of direct rheological, permeability, and biological measurements limit the extent to which long-term biomedical suitability can be established from these samples and tests alone. Future work should focus on controlled crosslinking, quantitative porosity and microstructural characterization, rheological and rate-dependent mechanical testing, and biological evaluation regarding application-specific formulations. The findings also support the need for standardized and reproducible characterization protocols for 3D printed hydrogels, particularly where different testing geometries can probe substantially different aspects of heterogeneous material behavior.

## Figures and Tables

**Table 1:** Five-stage development of 3D printing, including transition to bioprinting applications. Table reproduced from [12] under the terms of the Creative Commons Attribution 4.0 International (CC BY 4.0) License.

Stage	Description	Examples/Applications	Key Characteristics
I	Printing of medical models and instruments	Surgical models, in vitro devices	No interaction with the human body; no biocompatibility required
II	Permanent implants	Artificial hip, knee replacements	Biocompatibility required; degradability not necessary
III	Tissue engineering based on scaffolds	Cell growth scaffolds	Biocompatible, degradable, and absorbable materials
IV	3D bioprinting with living cells	Biomimetic tissues and organs	Direct printing of cells; regenerative medicine applications
V	Organoids and micro-organs	Microphysiological systems, in vitro organs	Complex living systems; advanced tissue models

**Table 2:** Overview of the main bioink types. Table reproduced from [12] under the terms of the Creative Commons Attribution 4.0 International (CC BY 4.0) License.

Type of Bioink	Description	Advantages	Challenges	Application	Recent Advances
Hydrogels	Water-rich, crosslinked polymers that mimic the ECM.	High biocompatibility and tunable properties.	Low mechanical strength, slow degradation.	Tissue engineering, wound healing, drug delivery.	Shear-thinning formulations for improved printability.
Decellularized ECM (dECM)	ECM-derived bioinks retaining native tissue structure.	Strong biological relevance and cell support.	Complex processing, possible immunogenicity.	Regenerative medicine, organ-specific constructs.	Improved decellularization preserving bioactivity.
Nanomaterial bioinks	Bioinks enhanced with nanoparticles or nanocomposites.	Improved mechanics and functional activity.	Toxicity risk, dispersion challenges.	Bone repair, drug delivery, antimicrobial systems.	Multifunctional nanomaterials for enhanced performance.
Cell aggregates	Spheroids or cell clusters that self-assemble into tissue.	Natural tissue formation and maturation.	Difficult handling and limited printability.	Organoids, tissue models, regeneration.	Bioreactors for scalable tissue maturation.
Synthetic polymers	Engineered polymers with controlled properties.	Tunable mechanics and degradation.	Limited bioactivity.	Hard tissue engineering, scaffolds.	Biodegradable polymers with adjustable degradation.
Natural polymers	Biopolymers such as collagen, gelatin, alginate.	Good biocompatibility and cell support.	Weak mechanics, batch variability.	Soft and vascular tissue engineering.	Improved crosslinking for stability.
Composite bioinks	Hybrid systems combining natural and synthetic materials.	Balanced mechanical and biological properties.	Complex formulation.	Multifunctional tissue engineering.	Nanomaterial- and bioactive-enhanced hybrids.

**Table 3:** Composition of the experimentally screened formulations.

Component	Initial Experimental Formulation	Refined Formulation	Final Formulation	Unit	Material/Source and Relevant Specification
Agar-based gelling blend	2.5	3.5	3.5	g	Eterika, Trstenik, Serbia; commercial agar-based gelling blend *
Omega-3 fish oil	4	4	4	μL	Fairvital, Karlsruhe, Germany; 1000 mg fish oil per softgel, containing EPA and DHA and supplemented with vitamin E *
Polysorbate 80 (Tween^®^ 80)	4	4	4	μL	Carl Roth GmbH + Co. KG, Karlsruhe, Germany; CAS No. 9005-65-6
Soy lecithin	-	4	4	μL	SojaVita, Belgrade, Serbia; commercial soy lecithin *
Glycerin preparation	4	4	4	μL	VZK Laboratory, Belgrade, Serbia; commercial glycerin preparation *
Psyllium husk powder (Plantago ovata)	-	0.4	0.5	g	We Are One, Belgrade, Serbia; product origin: India; mechanically milled and sieved before use
Ethanol solution, 70% (*v*/*v*)	2	2	2	μL	LIVSANE, BENU Pharmacy, Belgrade, Serbia; commercial 70% (*v*/*v*) ethanol solution *
L-Ascorbic acid	-	2	2	mg	Mivolis Vitamin C powder, dm-drogerie markt, Karlsruhe, Germany; pure L-ascorbic acid, pharmacopoeial quality
Hyaluronic acid (≈150 kDa)	0.03	-	-	g	In-house produced *
Demineralized water	10	10	10	mL	Denkmit, dm-drogerie markt, Karlsruhe, Germany; commercially sold as distilled water; demineralized water *

* Where analytical grade, purity, catalogue number, or detailed composition could not be verified from the available product documentation or laboratory records, no value was assigned. In such cases, the commercial product and supplier are reported, and the material was used as received.

**Table 4:** Printing parameters and sample configuration.

Sample ID	Speed (mm/s)	Infill (%)	Infill Pattern
S1	1	5	Lines
S2	1	15	Lines
S3	1	30	Lines
S4	2	5	Lines
S5	2	15	Lines
S6	2	30	Lines
S7	5	5	Lines
S8	5	15	Lines
S9	5	30	Lines

**Table 5:** Calculated theoretical values of mass and experimentally measured mass of samples.

Sample ID	Infill [%]	Calculated Mass Immediately After 3D Printing [g]	Mass Measured Immediately After 3D Printing [g]	Mass Measured After 3 Days [g]	Mass Measured After 7 Days [g]	Mass Measured After 110 Days [g]
S1	5	0.63	2.24	0.6971	0.688	0.6800
S2	15	1.89	7.0277	2.314	2.249	0.6308
S3	30	3.78	8.7561	2.7691	2.713	1.8538
S4	5	0.63	2.5231	0.7533	0.744	0.6879
S5	15	1.89	6.9521	2.1688	2.148	1.42
S6	30	3.78	10.0398	3.3948	3.241	1.584
S7	5	0.63	2.0508	0.7264	0.716	0.6045
S8	15	1.89	7.3389	2.4736	2.439	1.746
S9	30	3.78	9.7443	3.2996	3.168	2.9361

**Table 6:** Mass loss of samples after 3, 7, and 110 days, including differences between these time periods.

Sample ID	Infill (%)	Mass Loss, 3rd Day [%]	Difference, 0–3rd Day [%]	Mass Loss, 7th Day [%]	Difference, 3rd–7th Day [%]	Mass Loss, 110th Day [%]	Difference, 7th–110th Day [%]
S1	5	68.88	31.12	69.29	0.41	69.64	0.36
S2	15	67.07	32.93	68.00	0.92	91.02	23.03
S3	30	68.38	31.62	69.02	0.64	78.83	9.81
S4	5	70.14	29.86	70.51	0.37	72.74	2.22
S5	15	68.80	31.20	69.10	0.30	79.57	10.47
S6	30	66.19	33.81	67.72	1.53	84.22	16.50
S7	5	64.58	35.42	65.09	0.51	70.52	5.44
S8	15	66.29	33.71	66.77	0.47	76.21	9.44
S9	30	66.14	33.86	67.49	1.35	69.87	2.38

**Table 7:** Experimentally measured values of density 7 days after fabrication.

Sample ID	S1	S2	S3	S4	S5	S6	S7	S8	S9
Density g/cm^3^ 7th day	0.24	0.44	0.34	0.30	0.64	1.24	0.22	0.63	0.48

**Table 8:** Maximum compressive load achieved for each sample using a flat and a spherical indenter, together with the corresponding indentation depth.

Sample No.	S1	S2	S3	S4	S5	S6	S7	S8	S9
Printing speed [mm/s]	1	1	1	2	2	2	5	5	5
Infill [%]	5	15	30	5	15	30	5	15	30
Maximum load [N] at 3 mm depth (flat indenter)	70.22	16.13	9.61	19.76	161.81	174.66	9.12	183.58	9.71
Indentation depth [mm] (flat indenter)	2.98	3	2.99	2.71	3	3	2.2	2.99	3
Maximum load [N] (spherical indenter)	7.8	24.57	5.54	8.19	10.93	396.24	0.15	0.69	20.2
Indentation depth [mm] (spherical indenter)	2.21	2.61	1.59	1.8	2.92	3	2.97	2.1	2.92

**Table 9:** Comparison between maximum stress levels at compressive tests with spherical and cylindrical indenter: Fs—maximum load achieved with spherical indenter, experimentally obtained data; ε_r_—calculated representative strain with spherical indenter; σ_r_—calculated representative stress with spherical indenter; Ff—maximum load achieved with flat indenter, experimentally obtained data; σ_true_—calculated true stress with flat indenter; ε_true_—calculated true (logarithmic) strain with flat indenter.

Sample	Fs [N]	ε_r_ [–]	σ_r_ [MPa]	Ff [N]	True (Logarithmic) Strain ε_true_ [–]	σ_true_ [MPa]	Stress Ratio σ_r_/σ_true_ [–]
S1	7.8	0.083	0.040	70.22	0.35	0.0548	0.73
S2	24.57	0.091	0.107	16.13	0.36	0.0125	8.56
S3	5.54	0.071	0.040	9.61	0.36	0.0075	5.33
S4	8.19	0.075	0.052	19.76	0.32	0.0160	3.25
S5	10.93	0.096	0.043	161.81	0.36	0.1259	0.34
S6	396.24	0.097	1.505	174.66	0.36	0.1358	11.08
S7	0.15	0.097	0.001	9.12	0.25	0.0079	0.13
S8	0.69	0.081	0.004	183.58	0.36	0.1430	0.03
S9	20.2	0.096	0.079	9.71	0.36	0.0076	10.39

**Table 10:** Contact angle measurements and mean values for samples 1–9. θL and θR represent the contact angles measured on the left and right sides of each droplet, respectively. θLR is the mean of θL and θR for an individual droplet, while θavg is the mean of the three θLR values obtained for each sample.

Sample	Measurement	Left Contact Angle, θL (°)	Right Contact Angle, θR (°)	Mean Angle per Droplet, θLR (°)	Mean Contact Angle per Sample, θavg (°)
1	1	43.4	30.1	36.75	33.88
	2	24.3	31.0	27.65	
	3	30.6	43.9	37.25	
2	1	36.6	32.8	34.70	33.33
	2	24.6	40.0	32.30	
	3	28.6	37.4	33.00	
3	1	40.2	47.7	43.95	50.63
	2	61.9	65.9	63.90	
	3	41.1	47.0	44.05	
4	1	30.1	24.7	27.40	31.72
	2	34.6	29.2	31.90	
	3	34.8	36.9	35.85	
5	1	23.7	27.3	25.50	30.07
	2	32.7	33.2	32.95	
	3	32.8	30.7	31.75	
6	1	53.5	46.3	49.90	48.18
	2	36.1	43.9	40.00	
	3	58.2	51.1	54.65	
7	1	42.4	36.9	39.65	38.47
	2	32.8	39.1	35.95	
	3	36.9	42.7	39.80	
8	1	37.7	36.9	37.30	37.88
	2	36.9	32.2	34.55	
	3	39.8	43.8	41.80	
9	1	53.8	48.7	51.25	44.38
	2	30.3	38.4	34.35	
	3	39.4	55.7	47.55	

**Table 11:** Descriptive influence of printing parameters on the mean water contact angle. Group means were calculated from the three samples sharing each parameter level.

Printing Parameter	Level	Mean θavg (°)	Range Between Level Means (°)	Descriptive Influence
Infill	5%	34.69	13.97	More pronounced
	15%	33.76		
	30%	47.73		
Printing speed	1 mm/s	39.28	3.59	Less pronounced
	2 mm/s	36.66		
	5 mm/s	40.24		

**Table 12:** Classification of the samples according to mean water contact angle and relative wettability, ordered by the ranking.

Sample	θavg (°)	Surface Behavior	Relative Wettability	Rank
S5	30.07	Hydrophilic	Highest	1
S4	31.72	Hydrophilic	Very high	2
S2	33.33	Hydrophilic	Very high	3
S1	33.88	Hydrophilic	Very high	4
S8	37.88	Hydrophilic	Intermediate	5
S7	38.47	Hydrophilic	Intermediate	6
S9	44.38	Hydrophilic	Intermediate-lower	7
S6	48.18	Hydrophilic	Lower	8
S3	50.63	Hydrophilic	Lowest	9

## Data Availability

The datasets generated and/or analyzed during the current study are available from the corresponding author upon reasonable request.
